# Bulk *versus* surface charge recombination kinetics in polymer heterojunction nanoparticle photocatalyst dispersions

**DOI:** 10.1039/d6sc07200c

**Published:** 2026-10-27

**Authors:** Soranyel Gonzalez-Carrero, Jan Kosco, Chen Zhang, Michael Sachs, Teng Fei, Iain McCulloch, James R. Durrant

**Affiliations:** a Institute of Molecular Science, University of Valencia 46980 Paterna Valencia Spain soranyel.gonzalez@uv.es; b Department of Chemistry, Centre for Processable Electronics, Imperial College London London W12 0BZ UK; c King Abdullah University of Science and Technology (KAUST), KAUST Solar Center (KSC) Thuwal 23955-6900 Saudi Arabia; d University of Vienna, Faculty of Chemistry, Institute of Physical Chemistry Währinger Straße 42 1090 Vienna Austria; e Department of Chemistry, Chemistry Research Laboratory, University of Oxford Oxford OX1 3TA UK james.durrant@chem.ox.ac.uk; f Andlinger Center for Energy and the Environment, Department of Electrical and Computer Engineering, Princeton University Princeton NJ 08544 USA

## Abstract

Organic donor–acceptor heterojunction nanoparticles have emerged as promising photocatalysts for solar hydrogen production, combining efficient light harvesting, donor–acceptor charge separation, and the generation of long-lived charge carriers. However, the identity of the charge populations responsible for photocatalytic turnover remains unresolved. Here, we study photogenerated polarons in aqueous oligo(ethylene glycol)-functionalized IDTBT:IDTBR-based heterojunction nanoparticles using transient absorption spectroscopy (TAS) and photoinduced absorption spectroscopy (PIAS) to probe, respectively, the transient polarons generated under pulsed excitation and long-lived polarons that accumulate under continuous illumination. By directly correlating spectroscopic signatures with photocatalytic hydrogen evolution, we show that the steady-state PIAS response tracks photocatalytic activity, establishing long-lived accumulated charges as the reactive population governing photocatalytic turnover. Excitation-fluence studies further demonstrate that the contrasting TAS and PIAS responses originate from distinct trapped-charge populations, with decay times at matched charge densities being five orders of magnitude faster for TAS compared to PIAS. We attribute the exceptionally long lifetimes measured in PIAS to the synergistic effects of donor–acceptor charge separation, progressive trap filling under continuous illumination, and dielectric stabilisation provided by the aqueous glycol-functionalized environment, which together suppress charge recombination and enable long-lived charge accumulation under operating conditions. These findings identify *operando* PIAS as a powerful approach for probing catalytically relevant charge populations and provide mechanistic design principles for engineering organic semiconductor photocatalysts with enhanced solar fuel performance.

## Abstract 2

A comparison of charge dynamics under pulsed excitation and continuous illumination reveals distinct charge populations in organic heterojunction nanoparticle dispersions, with long-lived accumulated charges correlating with hydrogen evolution.

## Graphical abstract

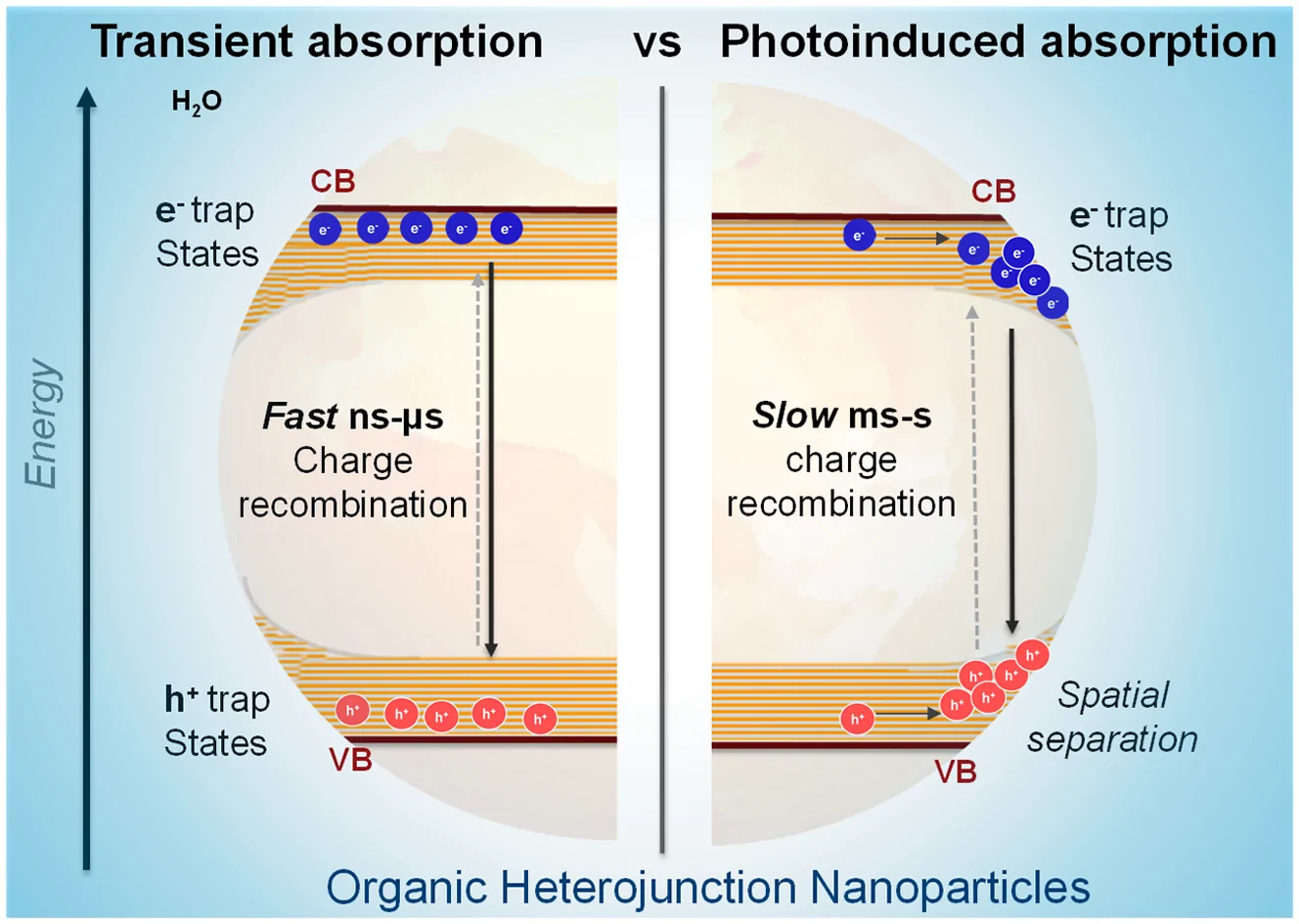

## Introduction

Converting solar energy into chemical fuels through photocatalytic water splitting offers a potentially sustainable and cost-effective route to hydrogen production. Despite significant progress on semiconductor material design, the overall efficiencies of photocatalytic particle-based devices remain significantly below those required for practical large-scale applications. The performance of particle photocatalytic systems is determined by fundamental processes, including light absorption, exciton/charge-carrier separation and transport, and interfacial charge transfer/redox catalysis.^1,2^ These processes extend over multiple timescales, from the picosecond to nanosecond lifetimes of bulk photoexcitations in most photocatalyst materials to the millisecond-to-second timescales of multi-redox catalysis. The efficiency of most photocatalytic systems is limited by charge recombination occurring either within the bulk or at the particle surfaces.^3^ As such, determining the kinetics of these recombination processes, their impact on photocatalytic performance, and how they can be suppressed through appropriate materials design are key challenges for the development of more efficient photocatalysts. In the study herein, we focus on charge dynamics in organic semiconductor photocatalyst nanoparticles and the insights that can be gained by comparing transient absorption studies under pulsed laser excitation with photoinduced absorption studies under one-sun-equivalent quasi-continuous irradiation.

Time-resolved spectroscopic techniques, particularly transient absorption spectroscopy (TAS), have been widely employed to probe charge generation, separation, trapping and recombination dynamics in particle photocatalyst dispersions, including metal oxides, conjugated polymers, metal–organic frameworks, and their heterojunctions, over timescales ranging from femtoseconds to seconds.^4,5^ TAS generally relies on short-pulse, high intensity, laser excitation to initiate the photophysical processes of interest. As such, whilst TAS has been invaluable in elucidating the underlying charge dynamics in photocatalyst particles, it has sometimes been challenging to quantitatively correlate the observed TAS kinetics with those under *operando* conditions using continuous, relatively low intensity sunlight irradiation. For example, non-linear processes such as bimolecular recombination can be greatly accelerated under pulsed laser excitation due to the instantaneous high photogenerated carrier densities relative to one-sun irradiation.^6^ Furthermore, the effects of photoinduced trap filling can be quite distinct under pulsed *versus* continuous irradiation.^7^ As such, elucidating charge-carrier dynamics under more *operando*, one-sun irradiation conditions is often a key challenge for the functional characterisation of solar conversion materials and devices.

Following photoexcitation, charge carriers in photovoltaic devices are generated and undergo relaxation, charge separation, and recombination on timescales ranging from femtoseconds to microseconds. Due to these short carrier lifetimes, only relatively low charge densities accumulate under one-sun irradiation, which are too low to be measurable in conventional TAS systems. Frequency-domain photoinduced absorption spectroscopy has been employed to overcome this sensitivity challenge using frequency-modulated excitation sources, however, extracting quantitative kinetic and population density information from such data is often challenging due to the more complex data analyses required.^8^ However, efficient photocatalysts or photoelectrodes generally require much longer-lived charges to drive interfacial catalytic reactions than those needed in photovoltaic devices. For example, the multi-redox water oxidation reaction typically proceeds on the millisecond-to-second timescale (ms–s) and therefore requires photogenerated charges (or, more correctly, oxidising equivalents) with lifetimes extending into this ms–s range.^9^ Such long charge lifetimes can lead to the accumulation of substantial photoinduced charge densities under one-sun irradiation. Indeed, for a range of photoelectrochemical and photocatalytic systems that drive water oxidation, we and others have demonstrated that one-sun-equivalent pulsed LED excitation (typically 5 s, quasi-continuous excitation) yields readily measurable photoinduced absorption signals from accumulated oxidising equivalents.^10,11^ For example, for SrTiO_3_ and BiVO_4_ photocatalyst particles, we have employed time-domain photoinduced absorption spectroscopy (PIAS) to quantitatively monitor the *operando* density of accumulated species under one-sun irradiation, the kinetics of water oxidation, and the impact of photoinduced trap filling, providing new insight into photocatalytic function.^12^

We have recently extended such time-domain PIAS studies to organic semiconductor photocatalyst nanoparticle suspensions based on conjugated donor/acceptor (D/A) heterojunctions. Such organic photocatalyst nanoparticles are attracting increasing interest for solar-driven hydrogen evolution under visible and near-infrared light irradiation.^13,14^ Strikingly, we observed that under one-sun irradiation, dispersions of organic D/A heterojunction nanoparticles in water, without added co-catalyst for proton reduction or an electron-donating species for hole extraction, produced long-lived (seconds timescale) PIAS signals, corresponding to the accumulation of up to 600 charges per nanoparticle.^15^ The density of these accumulated charges in neat nanoparticle dispersions was observed to correlate with enhanced photocatalytic activity for hydrogen production in the presence of added Pt co-catalyst and ascorbic acid as a hole scavenger.

The ability of organic D/A heterojunction dispersions to generate such long-lived charges depends critically on the water-nanoparticle interface, with negligible long-lived charge generation in dried films of the same nanoparticles.^16^ We and others have proposed that this long-lived charge generation results from the higher dielectric response of the aqueous electrolyte relative to the organic nanoparticle, causing an energetic stabilisation of charges on the nanoparticle surface relative to their bulk.^16,17^ However, these long (seconds) carrier lifetimes observed in our PIAS studies contrast with microsecond decay times in our transient absorption studies of the same particle suspensions. Moreover, TAS studies of these and other organic photocatalyst particles have indicated charge-extraction times to ascorbic acid and Pt of microseconds or faster. These observations suggest that second-timescale charge carrier lifetimes should not be necessary for efficient hydrogen evolution on such organic photocatalyst nanoparticles. As such, whilst the observation of long-lived charge accumulation in organic photocatalyst particle suspensions by PIAS is striking, its importance for achieving efficient photocatalytic performance has remained unclear.

Our observation that transient absorption studies of organic nanoparticles under short-pulse (typically ns) laser excitation in TAS can yield microsecond-timescale recombination, whilst long-pulse (typically seconds) LED excitation in PIAS yields milliseconds-to-seconds timescale recombination, is particularly surprising. A similar contrast in timescales between pulsed-laser and LED excitation has also been observed for inorganic photocatalyst particles, including those of SrTiO_3_ and BiVO_4_.^18,19^ Surprisingly, this distinction in recombination timescale appears not to result directly from differences in overall charge density, as this distinction in recombination timescale remains even for microsecond TAS and milliseconds-to-seconds PIAS data with similar signal magnitudes. As such, determining the origin of this distinction in the recombination timescale remains a key challenge for transient spectroscopic studies of photocatalyst particles.

In the study herein, we address the functional significance of long-lived charge accumulation in organic photocatalyst nanoparticles. To this end, we investigate charge dynamics under both pulsed-laser and continuous illumination in donor/acceptor (D/A) heterojunction nanoparticle photocatalysts, using a combination of transient absorption spectroscopy (TAS) and photoinduced absorption spectroscopy (PIAS), over timescales ranging from microseconds to seconds. As a model system, we selected a series of heterojunction NPs composed of the polymer donor indacenodithiophene–benzothiadiazole (IDTBT), blended with the non-fullerene acceptor indacenodithiophene (IDTBR), including modifications with polar oligo(ethylene glycol) side chains (OEG). In earlier work, we demonstrated that OEG side chain modifications in the polymer suppress exciton recombination and enhance charge-transfer state recombination, thereby increasing charge yield and extending lifetimes into the millisecond regime.^20^ Here, we build on these previous studies to exploring the effect of pulsed laser *versus* LED excitation conditions on charge-carrier dynamics in OEG-functionalized heterojunction NP dispersions and examine how these dynamics correlate with photocatalytic activity. By correlating these measurements, we reveal distinct relationships between transient and long-lived charge populations and establish which charge-carrier dynamics are most closely associated with photocatalytic turnover under operating conditions.

## Results and discussion

Fig. 1 shows the chemical structures of the conjugated polymer donor and molecular non-fullerene acceptor explored, which are modified either with alkyl side chains (IDTBT and oIDTBR) or oligoethylene glycol (OEG) side chains (gIDTBT and gIDTBR). These materials show HOMO and LUMO levels that are thermodynamically suitable for a type II heterojunction and for both ascorbic acid oxidation and proton reduction (Fig. 1b and S1). We explored the D/A heterojunction nanoparticles (NPs), 1:1 wt, IDTBT:oIDTBR, gIDTBT:oIDTBR, and, for comparison, the fully OEG-modified NP, gIDTBT:gIDTBR. The images of HRTEM revealed that all three heterojunction NPs exhibited a partial core–shell morphology (see Fig. S2), with the polymer donor IDTBT (or gIDTBT) preferentially located at the NP surface, and the average NP size was 70 ± 20 nm (see additional details in the SI). Further detail on nanoparticle synthesis and heterojunction nanomorphology has been reported previously.^20^

**Fig. 1:**
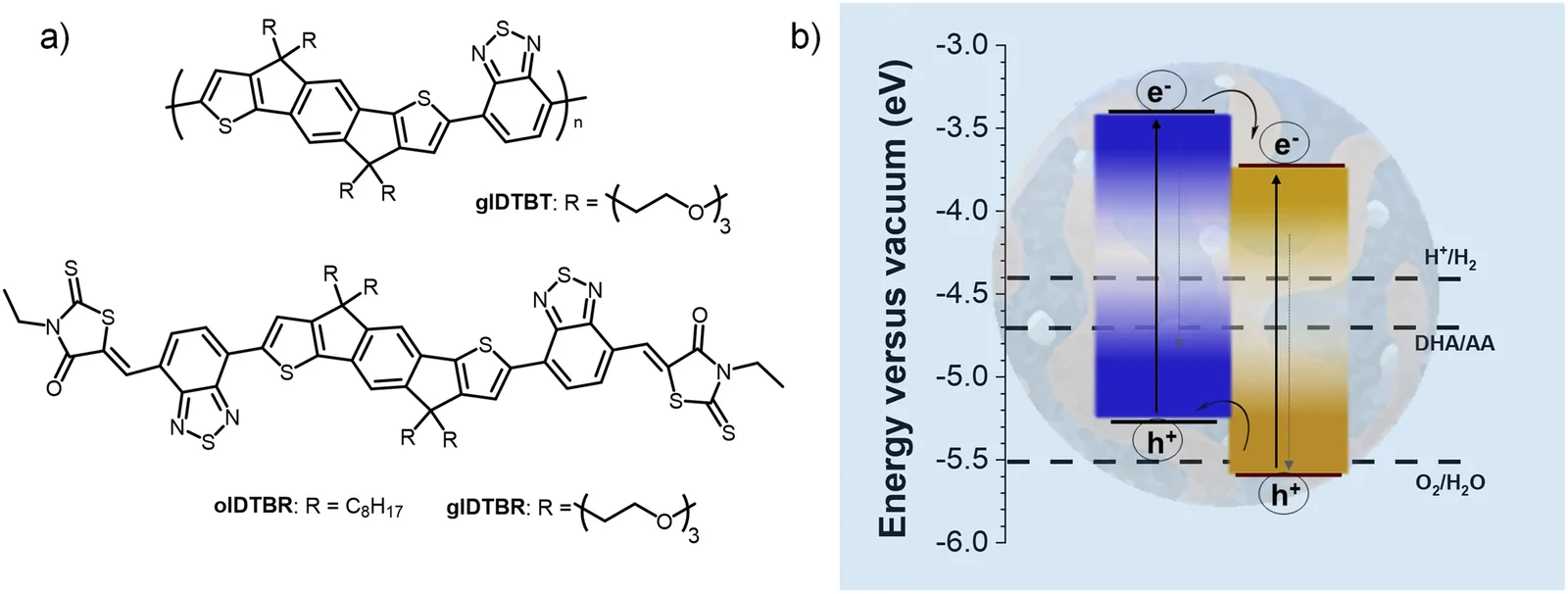
(a) Chemical structure of polymer donor IDTBT and the oIDTBR acceptor (g refers to OEG-side chains). (b) Schematic representation of the energy level diagram of a donor/acceptor NP interface showing a simplified viewpoint of photoexcitation. The diagram is illustrative and does not accurately represent the energy levels of the materials within the NPs.

The photocatalytic activity of NP dispersions for hydrogen production was previously reported using a Pt co-catalyst (10 wt%) and ascorbic acid (AA, 0.2 M) as a sacrificial electron donor.^20^ The hydrogen evolution rate (HER) was 15-fold and 30-fold larger in the gIDTBT:oIDTBR NPs compared to gIDTBT:gIDTBR and non-glycolated IDTBT:oIDTBR counterparts, respectively (see HER plot as reference in Fig. S3).

The D:A heterojunction NP dispersions showed a broad absorption spectrum in the visible with maximum absorption peaks at 400 and 650 nm, originating from π–π* transitions and intramolecular charge-transfer interaction between the D–A moieties along the polymer backbone, respectively (see absorption spectra and comparison with neat NP in Fig. S4–S6). Photoluminescence (PL) studies revealed strongly suppressed radiative recombination in the single-NP dispersion after OEG-side-chain replacement. Although the PL results alone do not provide conclusive evidence of charge generation, the observed suppression is consistent with charge generation previously demonstrated in glycolated IDTBT NP dispersion by ultrafast transient absorption spectroscopy (Fig. S7).^20^

### Charge recombination

We focus herein on the charge recombination dynamics of these heterojunction NPs dispersions on timescales ranging from microseconds to seconds in the absence of added sacrificial agents or added co-catalysts, under different irradiation conditions. First, we measured the transient absorption (TA) kinetics of heterojunction NP upon short, relatively high-intensity (6 ns) pulsed laser excitation (650 nm), which allows us to record microseconds–milliseconds decay times, as shown in Fig. 2. The heterojunction NP dispersions in water exhibit a broad photoinduced absorption spectrum with a dominant band centred at 1000 nm. This feature resembles previously reported ultrafast transient absorption spectra of IDTBT-based heterojunction NPs and is attributed primarily to the absorption of the IDTBT^+^ and oIDTBR^−^ polarons.^20,21^ Under identical conditions, the initial polaron amplitude is consistently higher in both OEG-heterojunction NP dispersions compared to their non-OEG counterpart (Fig. 2a and b), with recombination decay half-times (*t*_50%_ relative to *t*_0_ = 1 µs) of 10 µs for gIDTBT:gIDTBR and 5 µs for gIDTBT:oIDTBR.

**Fig. 2:**
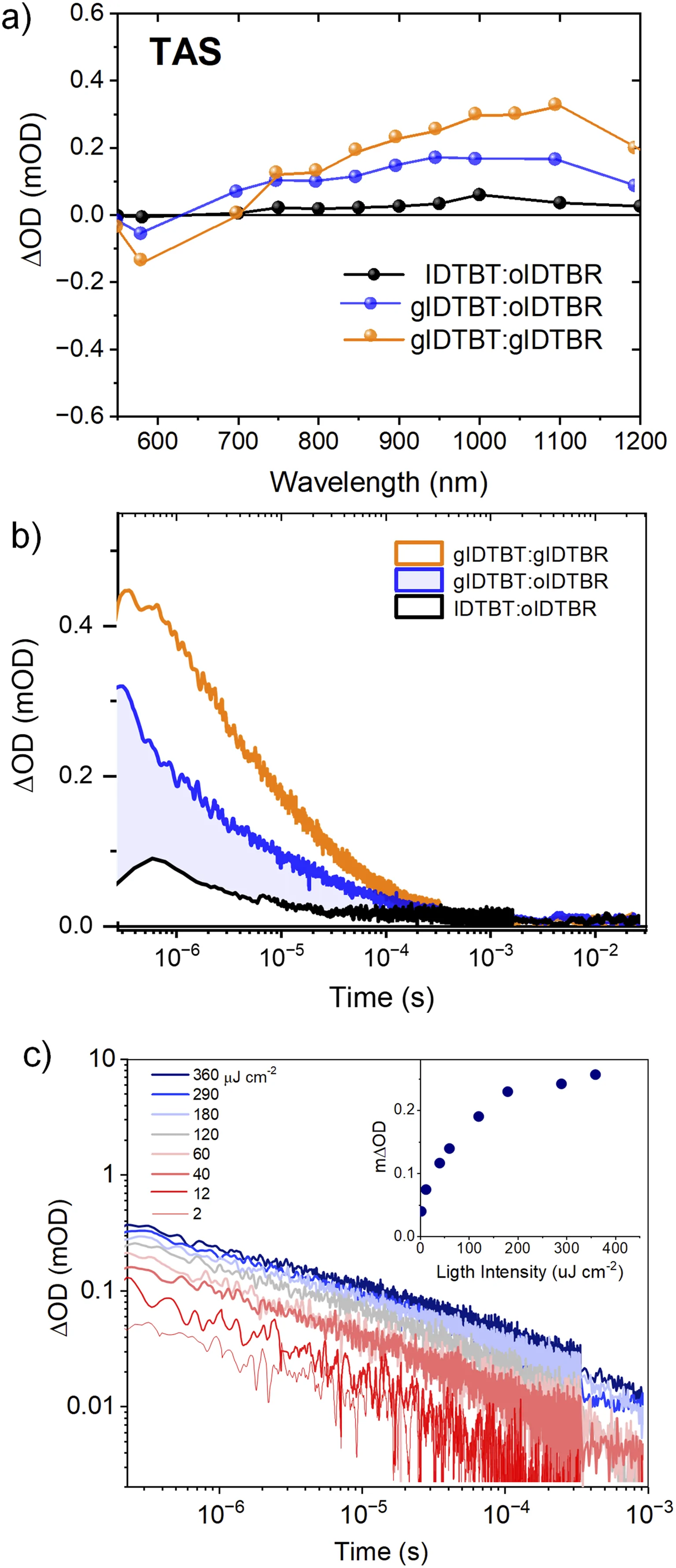
Comparison of (a) the TA spectra recorded at 2 µs time delay and (b) the corresponding TAS kinetics (probed at 1000 nm) of the D:A heterojunction NP dispersions. (c) TAS kinetics of gIDTBT:oIDTBR heterojunction NP at different light intensities. Inset: the optical signals (*m*ΔOD) as a function of light intensity. The NP dispersions were prepared in DI water under anaerobic conditions and adjusted to the same absorbed photon densities. Excitation wavelength was 650 nm.

A key focus of the study herein is the impact of optical excitation conditions on the observed recombination kinetics. Therefore, Fig. 2c plots the excitation density dependence of the TA kinetics for the gIDTBT:oIDTBR NPs, which exhibit the highest HER performance in our series. It is apparent that the TA decays in Fig. 2c are straight lines on the log/log axes employed, corresponding to power-law decay (ΔOD ∼ *t*^−*α*^) and indicative of bimolecular recombination. The *α* values obtained from the kinetic slopes of these decays (∼0.5) are indicative of dispersive bimolecular recombination. Such dispersive bimolecular recombination has been widely reported for organic bulk heterojunction (BHJ) films, and also, more recently for organic heterojunction NPs, and is assigned to trapping/detrapping limited bimolecular recombination with an exponential distribution of trap states, resulting in a broad range of decay times.^16^ Following the trapping/detrapping model *α* ∼0.5 corresponds to a characteristic energy for the exponential tail of trap states of 220 meV.^16,22^ Of particular relevance to the study herein, it is apparent that the decay dynamics are independent of excitation density. As discussed elsewhere for organic BHJ films,^23^ this implies that charges do not thermalise within the energetic distribution of traps prior to recombination, but rather primarily reside in the first trap (or traps) they encounter, prior to thermally driven de-trapping and recombination. This interpretation is also consistent with theoretical descriptions of dispersive trapping and detrapping in an energetic distribution of states, in which carriers initially populate traps of different energies and progressively redistribute toward deeper traps through detrapping-retrapping.^24^ As shallow trap occupancy decreases, the remaining trapped charges become increasingly dominated by deeper states, leading to slower detrapping and recombination kinetics.^24^ It is also apparent that the TA signal amplitudes in Fig. 2c increase sub-linearly with excitation density (see inset Fig. 2c), indicative of an excitation density-dependent faster recombination process, most likely corresponding to free carrier bimolecular recombination prior to charge trapping. In any case, it is apparent from these TAS data that the micro- to millisecond recombination dynamics of these NPs are independent of photoinduced charge density and indicative of trapping/detrapping-limited bimolecular recombination.

We now turn to examining the charge-recombination processes in the photoactive OEG-heterojunction NP dispersions over longer timescales (milliseconds to seconds) using photoinduced absorption spectroscopy (PIAS). This technique employs a low-intensity (circa 1 sun), long (quasi-steady state) LED excitation pulse, in contrast to the high-intensity nanosecond laser used in the TAS measurements described above. PIA has been used to assess photocatalyst efficiency by monitoring the accumulation of long-lived charges during water oxidation in materials such as metal oxides, carbon nitride, and conjugated polymers.^12,15^ In our measurements, a 630 nm LED (4 s, 20 mW cm^−2^) was used to excite the dispersions until reaching an equilibrium steady state between charge generation and recombination. After the LED was switched off, the accumulated charges decayed over milliseconds to seconds (Fig. 3). The resulting PIAS spectra, recorded at the maximum amplitude (average ΔOD), for all three heterojunction NP dispersions in water without sacrificial agents and under anaerobic conditions, are shown in Fig. 3a. Notably, only the OEG-heterojunction NPs exhibited clear PIAS signals, characterised by a ground-state bleach at 600 nm and a broad near-infrared photoinduced absorption centred at 1000 nm. Control PIAS measurements revealed larger absorption signals in the OEG-NP heterojunctions than in single-polymer or NFA NP dispersions (Fig. S8), supporting the assignment of the absorption features to enhanced charge separation across the donor–acceptor heterojunction. The PIAS spectral features of the OEG-heterojunctions closely resemble those observed in the TAS spectra (compare Fig. 2a and 3a), indicating that the long-timescale absorption also remains dominated by the IDTBT^+^ polarons. It is notable that gIDTBT:oIDTBR NPs exhibit both the largest PIAS signal and also the slowest PIAS decay kinetics upon LED off, with a decay halftime of circa 1 second (Fig. 3b), strikingly slower than the microsecond decay kinetics of the pulsed laser-induced transient absorption signals described above. In the absence of an added co-catalyst or scavenger, these slow PIAS decays are assigned primarily to the recombination of accumulated charges, although residual charge transfer processes cannot be ruled out.

**Fig. 3:**
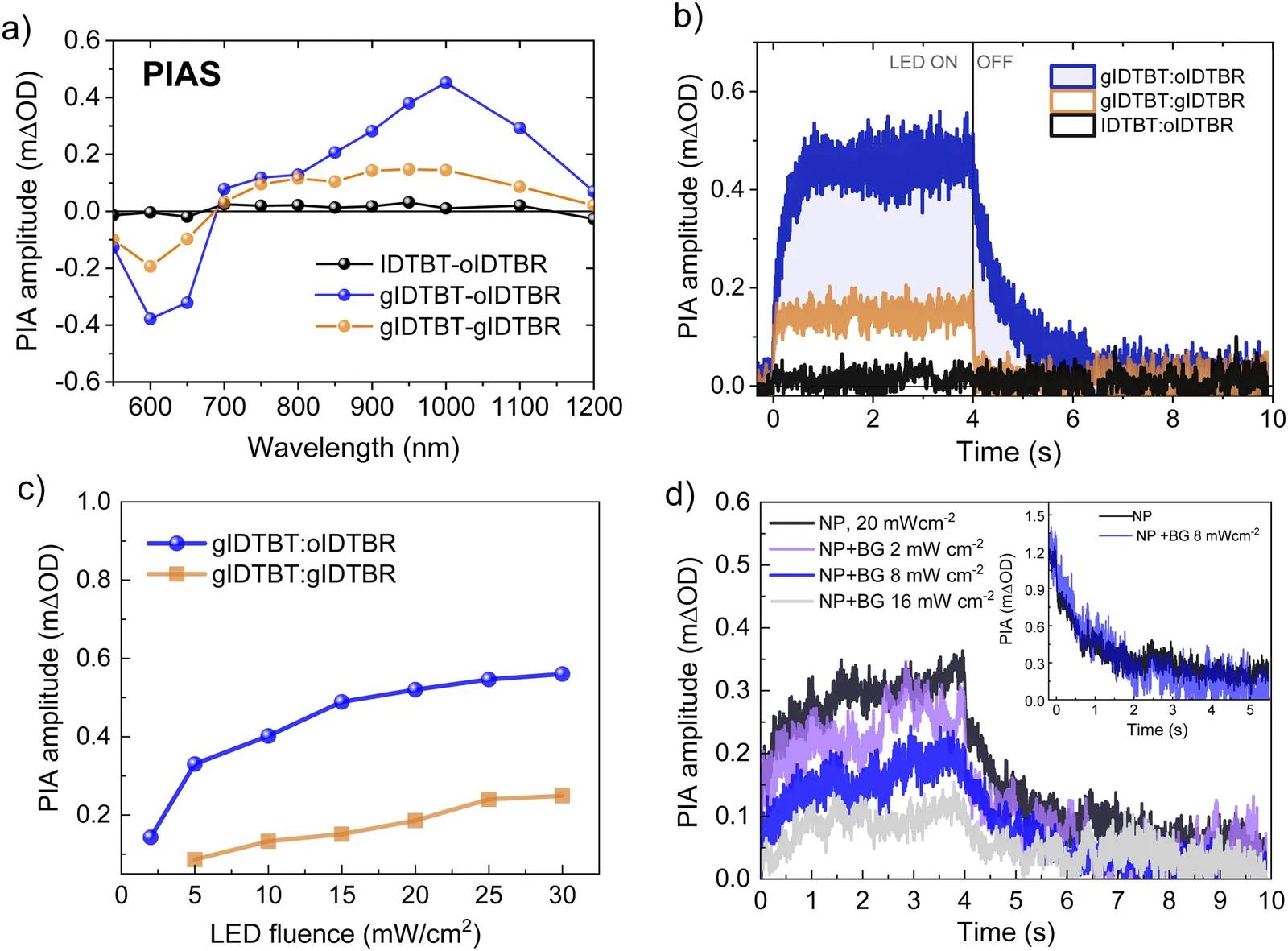
(a) Comparison of the PIAS spectra of D:A heterojunction NP dispersion in water under anaerobic conditions, recorded after 4 seconds of continuous LED illumination (630 nm, 20 mW cm^−2^). (b) Comparison of PIAS kinetics (probed at 1000 nm) of the heterojunction NP dispersion, assigned to IDTBT^+^ polaron absorption. (c) PIAS amplitude (*m*ΔOD average, probed at 1000 nm) of heterojunction NP dispersion at different LED fluences. (d) PIAS kinetics of gIDTBT:oIDTBR NP dispersion (probed at 1000 nm) recorded at 20 mW cm^−2^ (black trace) and additional (2, 8, and 16 mW cm^−2^) continuous 630 nm background illumination. Inset: comparison of normalised PIAS decay kinetics with/without additional BG illumination (8 mW cm^−2^).

We turn now to consideration of the impact of the excitation conditions upon the PIAS decay kinetics for gIDTBT:oIDTBR and gIDTBT:gIDTBR NPs. Fig. 3c plots the PIAS transients of these heterojunction NPs under different LED fluences, reaching saturation above approximately 20 mW cm^−2^ for gIDTBT:oIDTBR, whereas gIDTBT:gIDTBR NPs exhibit a more gradual, nearly linear increase up to approximately 25 mW cm^−2^. This difference in saturation behaviour most likely results from the more efficient long-lived charge generation observed for gIDTBT:oIDTBR NPs at all LED intensities. Despite the increase in PIAS signal and, therefore, accumulated charge density, the decay kinetics for both NP dispersions remained fluence-independent across all excitation conditions (Fig. S9). Additional measurements under continuous background illumination showed that the PIAS amplitude decreased by up to 60% under this background excitation (Fig. 3d), whereas again the changes in decay kinetics were negligible (inset Fig. 3d). The saturation of PIAS amplitude with increasing LED intensity and the reduction in PIAS amplitude under background irradiation are both evidence for photoinduced charge accumulation suppressing the yield of long-lived charges, indicative of faster, shorter timescale recombination processes, as we discuss further below. The independence of PIAS decay kinetics on light irradiation intensity is more striking and suggests that the decay dynamics of accumulated charges under photoirradiation are independent of the density of accumulated charges.

The striking contrast between the charge lifetimes observed in our TAS and PIAS studies is illustrated in Fig. 4 for gIDTBT:oIDTBR NPs (see Fig. S10 for additional NPs). Excitation densities were selected to ensure nearly matching initial signal amplitudes (and therefore average charge densities) between the two measurements. It is apparent that the PIAS decay kinetics are 5 orders of magnitude slower than TAS decay kinetics measured for the same photogenerated charge densities. As the TAS and PIAS decay kinetics are essentially independent of excitation density (Fig. 2c, 3d and S9), this remarkable difference between TAS and PIAS decay kinetics is independent of the excitation densities used (exemplar data at the lowest excitation density is shown in Fig. S11). Given this substantial difference in charge carrier lifetimes, it is clear that the two measurements result in the generation of different charge populations. As we discuss below, we will assign this difference in dynamics to our TAS studies which primarily probe bulk charge recombination, and our PIAS studies which primarily probe surface charges.

**Fig. 4:**
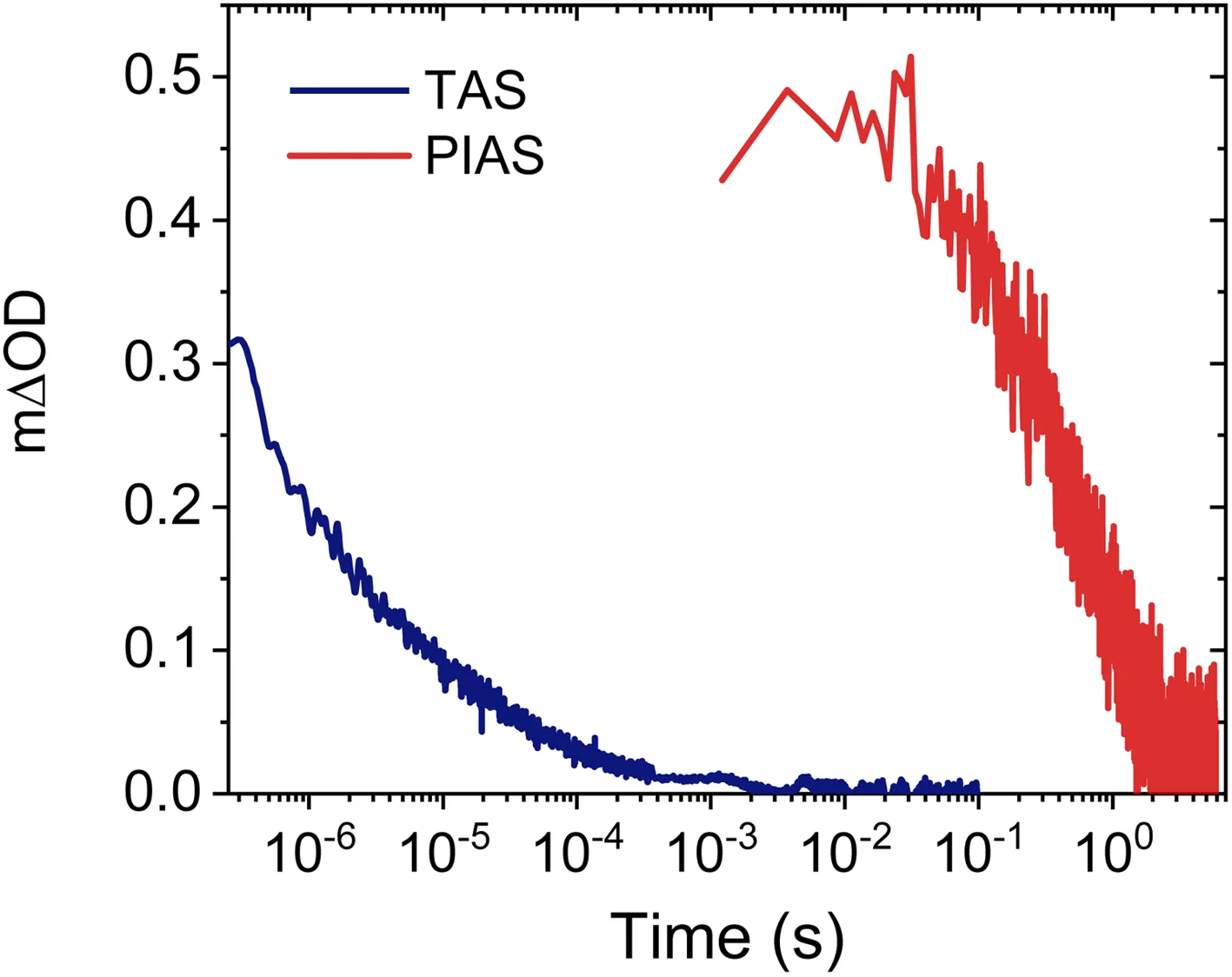
Comparison of TAS and PIAS traces (probed at 1000 nm) of gIDTBT:oIDTBR NP dispersion in water under anaerobic conditions under excitation conditions selected to give approximately matched densities of charge carriers at the start of each measurement. TAS: pulsed excitation at 650 nm (290 µJ cm^−2^). PIAS:LED illumination at 630 nm (20 mW cm^−2^).

Finally, we consider the relationship between our PIA and TAS data and *operando* HER performance, measured in a dispersion of NPs with 10 wt% Pt as a cocatalyst and 0.2 M AA as a sacrificial electron donor. TAS measurements showed faster polaron decay in the presence of AA or Pt-AA, consistent with sub-microsecond hole and hole/electron transfer processes (Fig. S12). Under these conditions, the PIAS measurements showed a ∼90% decrease in the absorption amplitude across the spectra in the presence of Pt/AA (Fig. S13). This substantial PIAS quenching is consistent with fast charge extraction and recombination *via* electron and hole transfer to Pt and AA, respectively, in agreement with the TAS kinetics. However, what is most striking is the consideration of the correlations between our TAS and PIAS signal amplitudes, measured without AA or Pt, and overall HER performance, as plotted in Fig. 5. It is apparent that our TAS initial signal amplitudes do not track the measured hydrogen evolution rates, with gIDTBT:gIDTBR showing the largest TAS signal, but gIDTBT:oIDTBR the highest HER rate (see also Fig. 2 TAS kinetics amplitude). In contrast, the PIAS amplitudes follow the same activity trend, gIDTBT:oIDTBR > gIDTBT:gIDTBR > IDTBT:oIDTBR. Whilst limited to only three samples, this matching of HER and PIAS trends is analogous to that we have reported previously for PM6 based BHJ NPs and suggests that our PIAS assay of the density of accumulated long-lived charges is a better predictor of hydrogen evolution efficiency than TAS studies measured using pulsed laser excitation.

**Fig. 5:**
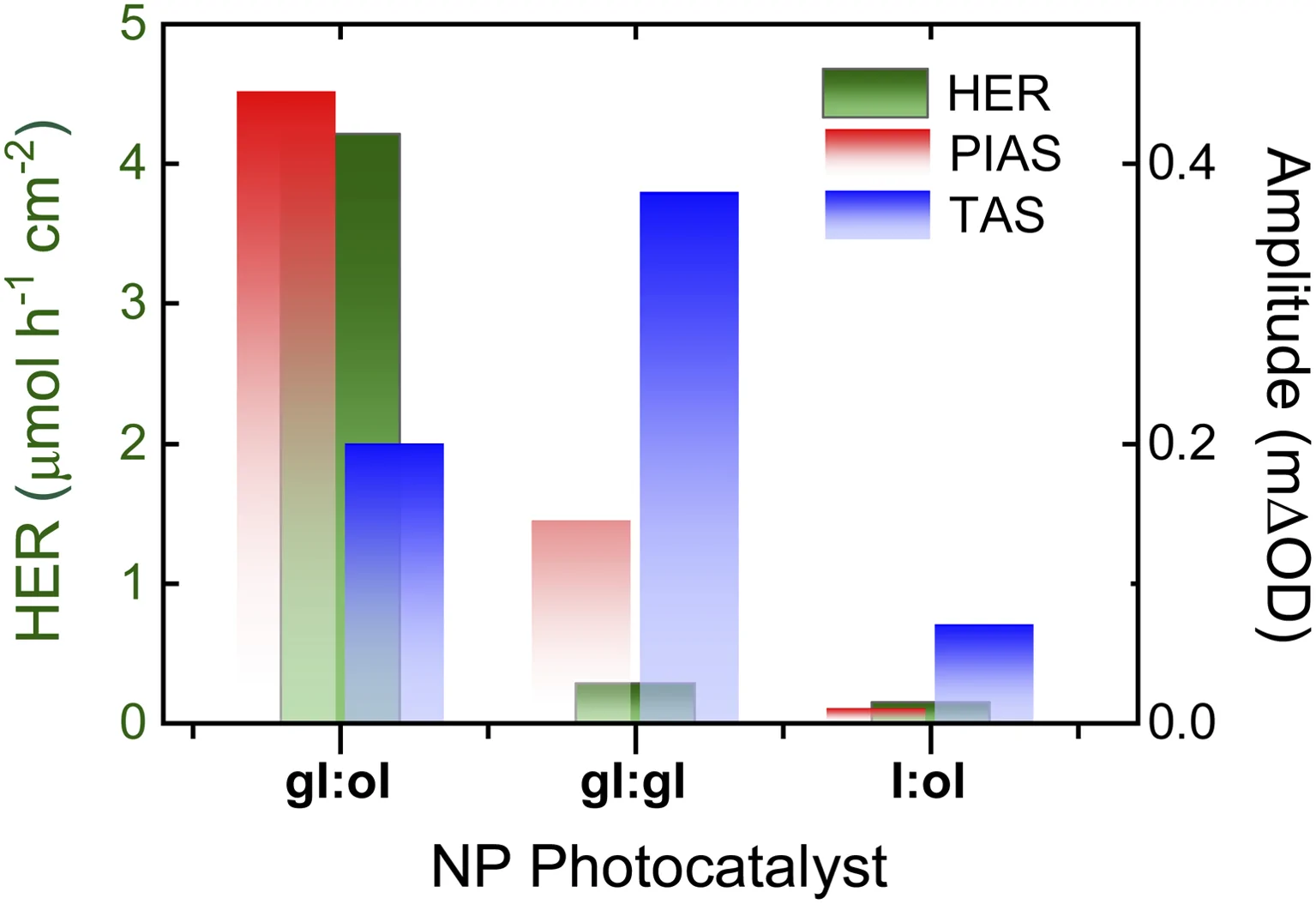
Comparison of TAS and PIAS kinetic amplitudes (*m*ΔOD probed 1000 nm) with the maximum average hydrogen evolution for the heterojunction NP order gIDTBT:oIDTBR (gI:oI), gIDTBT:gIDTBR (gI:gI) and IDTBT:oIDTBR (I:oI). The amplitude was measured from TAS kinetics at 2 µs, and PIAS kinetics were averaged over 4 seconds. The average hydrogen evolution rates of D:A heterojunction (1:1 wt.) at 16 h, measured using 1 mg nanoparticles, ascorbic acid (0.2 mol L^−1^, 12 mL, pH 2), Pt (10 wt%), AM1.5G solar simulator (100 mW cm^−2^; 1 sun), and a 4.41 cm^2^ illumination area.

## Discussion

In this work, we investigated heterojunction NPs composed of the OEG-modified polymer IDTBT and the non-fullerene acceptor IDTBR. Transient absorption (TAS) and photoinduced absorption spectroscopy (PIAS) measurements on their aqueous dispersions reveal the formation of IDTBT^+^ polarons. The two techniques, however, probe markedly different recombination regimes. PIAS probes charge populations persisting into the second timescale, whereas TAS captures recombination dynamics predominantly within the µs–ms regime. Furthermore, only the steady-state PIAS amplitudes correlate with photocatalytic hydrogen evolution activity. We now discuss the origin of these charge-recombination features, considering four potential factors: (i) the nature of the heterojunction material, (ii) the excitation fluence effects on charge dynamics, (iii) the role of scavengers, and (iv) the influence of the aqueous environment on the NP dispersion dynamics.

### Nature of the heterojunction material

Previous studies have demonstrated that the photocatalytic activity of D/A heterojunction nanoparticles depends strongly on the donor–acceptor interactions, blend composition, and resulting internal nanostructure, all of which influence charge separation, transport, and recombination pathways. Because these structural and morphological parameters can vary substantially between different heterojunction systems, direct mechanistic comparisons are inherently complex. Nevertheless, to assess whether the differences in charge behaviour observed herein between TAS and PIAS studies are a more general characteristic of photo-catalytically active heterojunction NP dispersions, we compared the charge dynamics across a range of active D/A NP systems prepared with different material combinations and blend ratios. Consistent with the trends described above for glycolate-modified NPs, heterojunction NP dispersions that exhibit measurable hydrogen evolution activity, such as PM6- and PTB7-TH-based heterojunctions, display large PIAS amplitudes and comparatively slower decay dynamics (ms–s) than those recorded in TAS in the absence of added scavengers (Fig. S14). We note we have also reported an analogous correlation between PIAS amplitude and HER performance in polymer donor/dimerized acceptor NP photocatalysts.^25^ These observations suggest that the generation and stabilization of long-lived charge populations, as assayed by PIAS studies are strong indicators for efficient photocatalytic activity in donor–acceptor nanoparticles. However, as discussed below, photocatalytic performance depends not only on charge lifetime but also on the accessibility and reactivity of these accumulated charges.

### Effect of the excitation density on recombination dynamics

On ultrafast timescales, exciton separation across the donor/acceptor interface results in the generation of free electron and hole polarons. Initially, these charges undergo relatively fast (ps–ns) bimolecular recombination which accelerates with increasing excitation density, as for example we have reported previously for gIDTBT:oIDTBR NPs.^20^ This ultrafast free charge recombination is in kinetic competition with charge trapping into shallow trap states. These shallowly trapped carriers subsequently undergo relatively slow (ns–ms) dispersive bimolecular recombination associated with thermally activated detrapping.^22,24,26^ Such trapping/detrapping limited recombination is also observed in a range of other photocatalytic materials, including metal oxides and carbon nitrides.^12,27^ In the OEG-heterojunction NP dispersion explored, TAS studies at different excitation fluences reveal fluence-independent power-law decay kinetics, consistent with trap-assisted recombination of dissociated charge carriers relaxing through an exponential distribution of localised trap states, as previously reported for PM6:PCBM heterojunction NP dispersions. The insensitivity of the TA decay kinetics to excitation density suggests that the faster µs-recombination observed in TAS cannot be attributed only to higher average photogenerated charge densities. Similar excitation and, therefore, charge density independence were also observed in PIAS studies. Moreover, similar initial signal sizes, and therefore charge densities, were observed in the TAS and PIAS studies (Fig. 2 and 3). These observations therefore indicate that variations in excitation density alone cannot determine the contrasting recombination kinetics observed by TAS and PIAS. Instead, they suggest that both probe intrinsically distinct recombination regimes and charge populations, rather than simply different charge-density limits.

### Effect of the scavenger

TAS kinetics in the presence of AA and Pt-AA were faster than those of NP dispersions in water, indicating efficient interfacial charge transfer on the sub-microsecond/microsecond timescale. Under *operando* conditions, however, the PIAS amplitude decreased by ∼90%, consistent with rapid charge extraction by the cocatalyst and sacrificial donor. These observations indicate that the long-lived charges detected by PIAS in the absence of scavengers correspond to an accumulated charge population. Although TAS resolves the initial charge-transfer processes, the TAS signal amplitudes do not correlate with photocatalytic activity. In contrast, the steady-state charge accumulation probed by PIAS shows a direct correspondence with hydrogen evolution, suggesting that these accumulated charges are more closely associated with the catalytically active population.

### Influence of the aqueous environment on NP dispersions

Previous studies have shown that D/A heterojunction NPs dispersed in water and their corresponding thin films exhibit comparable photogenerated charge-carrier dynamics on ultrafast timescales (<6 ns), indicating that the initial charge generation and bimolecular recombination processes are largely unaffected by water exposure.^16^ In contrast, pronounced differences emerge at longer timescales, with heterojunction NPs in aqueous dispersion exhibiting significantly longer charge-carrier lifetimes than thin films. This behaviour has been attributed to the interaction between the NP surface and the highly polar aqueous environment, which enhances the dielectric stabilisation of separated charges and facilitates the accumulation of long-lived charge populations.^16^ As such, the surrounding aqueous medium plays a critical role in charge recombination in organic semiconductor nanoparticles.

Our PIAS results demonstrate improved charge accumulation in OEG-heterojunction NPs. The remarkably slow recombination observed on the second timescale in our PIAS studies can be attributed to both the spatial separation of charges by the D–A architecture and the additional dielectric stabilisation of surface-trapped charges provided by OEG side chains that interact with the aqueous environment. The role of OEG appears to be closely linked with the impact of water, as OEG-modified NPs measured as dry films show substantially reduced PIAS amplitudes (Fig. S15), most likely from the OEG modification making the NPs more hydrophilic and thus enhancing water driven dielectric stabilisation.

### Origin of distinct charge dynamics measured under pulse laser *vs.* LED excitation

Our discussion above highlighted the key impacts of donor/acceptor heterojunctions, charge trapping, and water-driven dielectric stabilisation on charge-recombination dynamics in organic NP dispersions. We conclude by proposing a simple model, based on these considerations, that can explain the contrasting timescales of TAS and PIAS decays and their relevance to *operando* photocatalytic function.

Under the 10 ns pulsed laser excitation employed in our TAS studies herein, a high density of photogenerated charges is generated simultaneously within each NP. This high density of photogenerated free charges results in rapid (initially ps–ns) bimolecular bulk recombination, in kinetic competition with charge trapping. This is consistent with our observation that our micro-second TAS signal amplitudes saturate at higher laser intensities, due to greater dominance of ps–ns carrier recombination limiting the yield of trapped carriers, as well as saturation of available bulk trap states. The resultant high density of trapped charges generates our observed µs–ms dispersive charge recombination. Due to the relatively short timescales of these recombination processes, charges may not have the time to find the lowest energy trap states, which, as discussed above, are primarily on the NP surface due to the additional stabilisation induced by the water dielectric response. As such, our TAS studies predominantly probe the recombination processes of shallowly trapped charges in the nanoparticulate interior-bulk recombination, as illustrated in Fig. 6a.

**Fig. 6:**
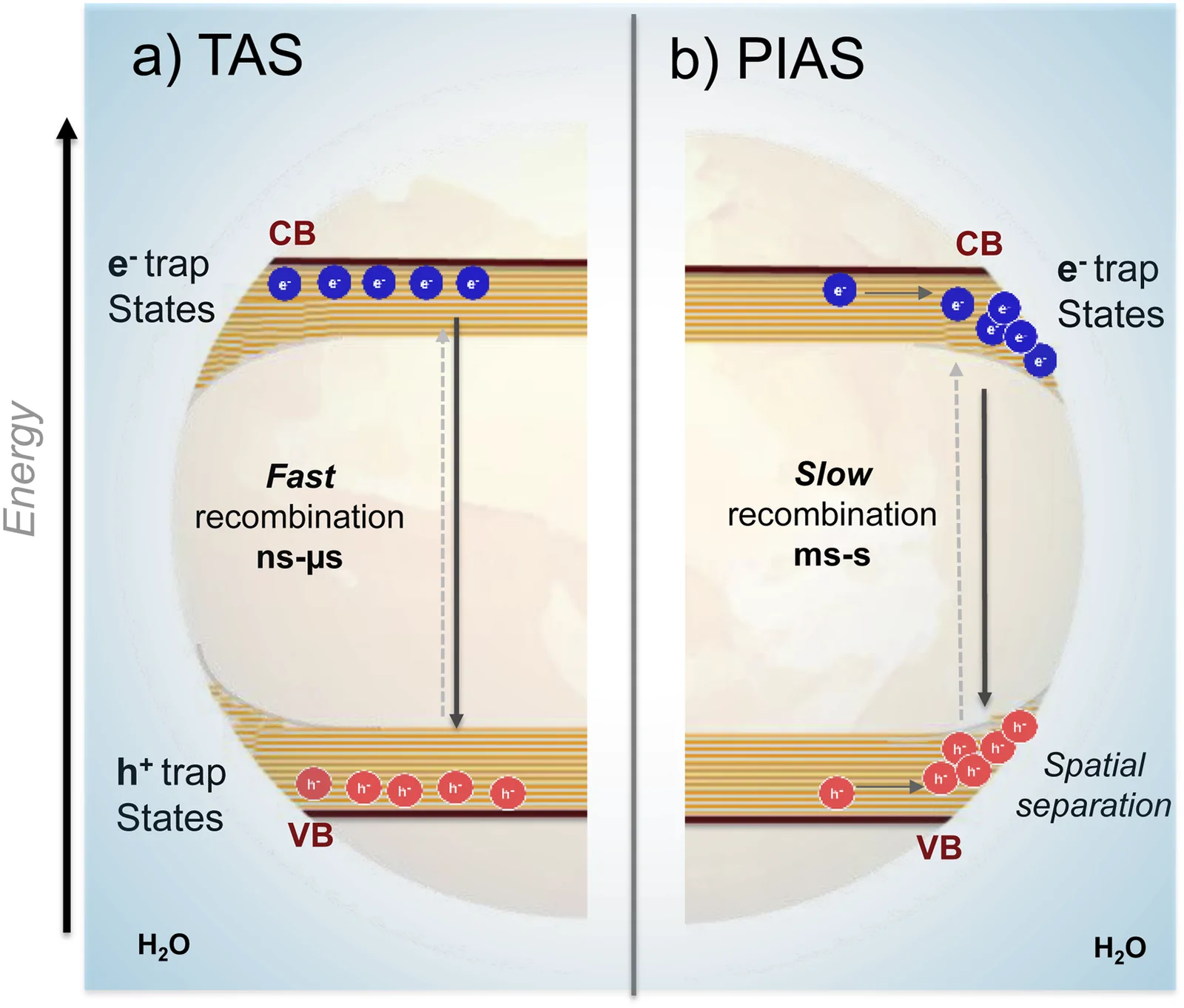
Schematic representation of trap state filling in heterojunction NP dispersion in water upon (a) high light flux used in TAS, and (b) quasi-continuous LED light excitation used in PIAS. For simplicity, the illustration does not explicitly represent the potential role of glycolation in facilitating water penetration into the nanoparticles or the presence of the donor–acceptor heterojunction responsible for exciton-charge separation.

In contrast, our PIAS studies employ quasi-continuous low-intensity illumination, of similar intensity to solar irradiation. Under these conditions, photogenerated charge densities in the NP bulk will be much lower, such that both free and trapping/detrapping mediated bimolecular recombination will be much slower than that occurring under pulsed laser excitation. In this case, photogenerated charges have sufficient time to diffuse further and reach the lowest-energy traps, lying primarily at the NP surface, as illustrated in Fig. 6b. This additional charge trapping, resulting from water driven dielectric relaxation, localizes and stabilises charges on the nanoparticle surface, maximizing the spatial separation of electrons and holes and substantially prolonging their lifetimes. We note that glycolation is likely to increase the penetration of water into the nanoparticle interior, increasing the effective NP ‘surface’ for such dielectric stabilisation.

Beyond the timescale difference, the contrasting decay profiles observed in TAS and PIAS further support distinct charge-decay mechanisms. The power-law decay observed in TAS is consistent with dispersive recombination across a heterogeneous distribution of trap states, involving thermally activated detrapping and a broad range of recombination timescales. In contrast, the approximately exponential PIAS decay kinetics most likely correspond to a surface recombination process, although other processes such as recombination *via* residual Pd ions or reactions with the electrolyte cannot be excluded. In any case, the difference in decay profiles supports the proposed distinction between predominantly bulk-associated recombination of shallowly trapped charges under pulsed excitation and the accumulation of long-lived, surface-stabilised charges under continuous illumination.

Taken together, our results establish a clear mechanistic picture: TAS predominantly probes earlier-time, high-density charge populations undergoing bulk recombination pathways, whereas PIAS under continuous illumination selectively probes the accumulation of long-lived surface-stabilized charges. As surface charges are critical to both hole extraction and proton reduction catalysis, this model is consistent with the PIAS showing a better correlation with photocatalytic efficiency than TAS. Consistent with this conclusion, the quantitative correlation between PIAS amplitude and hydrogen evolution rate across the heterojunction systems studied underscores that stabilisation of photogenerated charges on the NP surface is a key driver of photocatalytic performance.

The discussion above has focused on organic BHJ nanoparticles. However, it is striking that an analogous difference in TAS and PIAS decay times has been observed for inorganic photocatalyst particles, including BiVO_4_ and SrTiO_3_,^18,28^ with TAS decay times being orders of magnitude faster than PIAS decay times. It appears likely that, as for the organic NPs studied herein, this difference in decay times results from TAS data being dominated by bulk recombination and PIAS data being dominated by surface recombination. However, we note that for these inorganic photocatalysts, the difference in timescale between bulk and surface recombination most likely does not result from water driven dielectric stabilisation (as these metal oxides have similar or higher dielectric constants than water), but rather from surface band bending associated with Fermi level equilibration and/or surface dipoles from crystal lattice termination. We also note that this remarkable distinction between TAS and PIAS decay times observed for photocatalyst particles is observed to be less significant for metal oxide photoelectrodes. For example, surface holes driving water oxidation on hematite photoanodes exhibit similar (circa 1 s) decay times under both pulsed laser excitation (TAS) and long LED excitation (PIAS).^8^ This is most likely due to the strongly n-doped nature of most photoanodes, such that bulk hole lifetimes are very short, with both micro- to millisecond TAS and PIAS being dominated by holes that reach the surface space charge layer. In any case, it is apparent that the excitation conditions employed in PIAS studies are closer to *operando* solar irradiation than in TAS studies employing pulsed laser excitation, and that for photocatalyst particles in particular PIAS is best suited to probing the accumulation of long-lived surface charges critical to driving photocatalytic function.

## Conclusions

In summary, we combined TAS and PIAS to elucidate distinct charge-recombination pathways in donor–acceptor heterojunction nanoparticle dispersions. TAS, under pulsed laser excitation, probes early-time charge recombination dynamics on the µs–ms timescale, whereas PIAS, under continuous illumination, detects the accumulation of long-lived, surface stabilised charges on the second timescale. In the heterojunction NP systems investigated, the steady-state PIAS amplitudes correlate most clearly with photocatalytic hydrogen evolution activity, demonstrating that the stabilisation and accumulation of long-lived charges, rather than the initial charge-generation dynamics alone, influence photocatalytic performance. Excitation fluence studies further show that the contrasting TAS and PIAS responses cannot be explained by differences in charge density but instead arise from distinct trapped-charge populations and recombination pathways. The exceptionally slow recombination observed in aqueous OEG-functionalized heterojunction NPs is attributed to the combined effects of donor–acceptor charge separation, progressive trap filling under continuous illumination, and surface dielectric stabilization provided by the aqueous environment and OEG side chains. These findings establish PIAS as a valuable probe of long-lived reactive charge populations and underscore the critical role of charge stabilization in governing photocatalytic performance.

## Author contributions

S. G.-C. designed and conducted the spectroscopic measurements, performed formal analysis, and wrote the manuscript. J. K. synthesized the nanoparticle photocatalyst and contributed to the discussions. C. Z. and T. F. contributed to the spectroscopic measurements. M. S. contributed to the design of the spectroscopic experiment and data analysis. I. M. and J. R. D. supervised the project. All authors reviewed the manuscript.

## Conflicts of interest

There are no conflicts to declare.

## Supplementary Material

SC-OLF-D6SC07200C-s001

## Data Availability

The data supporting the findings of this study are available in the main article and the supplementary information (SI). Supplementary information: experimental details and methods, transmission electron microscopy studies, additional absorption and emission spectra, and transient and photoinduced absorption kinetics. See DOI: https://doi.org/10.1039/d6sc07200c.
