# Supplementary material for: Bulk versus surface charge recombination kinetics in polymer heterojunction nanoparticle photocatalyst dispersions
Source: Chem Sci. 2026 Oct 27. Online ahead of print. doi: 10.1039/d6sc07200c (PMC13728860; doi:10.1039/d6sc07200c)
Supplement: Supplementary file 1 [file SC-OLF-D6SC07200C-s001.pdf]

Supplementary Information (SI) for Chemical Science.
This journal is © The Royal Society of Chemistry

BULK VERSUS SURFACE CHARGE RECOMBINATION KINETICS IN POLYMER HETEROJUNCTION NANOPARTICLE PHOTOCATALYST DISPERSIONS

Soranyel Gonzalez-Carrero,*[a,b] Jan Kosco,[c] Chen Zhang,[b] Michael Sachs,[b,d] Teng Fei,[b] Iain McCulloch,[e,f] and James R. Durrant*[b,e]

[a] Institute of Molecular Science, University of Valencia, 46980 Paterna, Valencia, Spain

[b] Department of Chemistry and Centre for Processable Electronics, Imperial College London, London W12 0BZ, United Kingdom.

[c] King Abdullah University of Science and Technology (KAUST), KAUST Solar Center (KSC), Thuwal, 23955-6900, Saudi Arabia.

[d] University of Vienna, Faculty of Chemistry, Institute of Physical Chemistry, Währinger Straße 42, 1090 Vienna, Austria.

[e] Department of Chemistry, Chemistry Research Laboratory, University of Oxford, Oxford, OX1 3TA, United Kingdom

[f] Andlinger Center for Energy and the Environment, and Department of Electrical and Computer Engineering, Princeton University, Princeton, NJ, 08544, USA

Supporting information.

Materials.

Preparation of heterojunction nanoparticle dispersions. All chemicals were used without purification. Glycolated polymer gIDTBT and the small molecule acceptor gIDTBR were synthesized using our previously reported procedure.[1] The preparation of donor:acceptor (1:1 wt.) heterojunction NP dispersion was previously reported using the mini-emulsion method with sodium dodecyl sulfate (SDS) as the surfactant.[1] Briefly, gIDTBT:oIDTBR NP dispersion was prepared by mixing 2.5 mL of a gIDTBT stock solution (0.5 mg mL^{-1} in chloroform) with 2.5 mL of an oIDTBR stock solution (0.5 mg mL^{-1} in chloroform), yielding 5 mL of a precursor solution containing 1.25 mg of each component. This precursor solution was added dropwise to 10 mL of SDS solution (0.5 wt. %) under vigorous stirring at 40 °C for 15 min to form a pre-emulsion. The pre-emulsion was then sonicated for 5 min using an ultrasonic processor (Sonics VibraCell VCX130PB). Then, the chloroform was removed by heating the emulsion at 85 °C under an airstream, yielding a surfactant-stabilized NP dispersion in water. The final dispersion volume was adjusted to 7.5 mL with Milli-Q water.

The size and morphology of the heterojunction NP (1:1 wt.) were characterised by combining dynamic light scattering (DLS, Malvern Zetasizer ZS) and high-resolution transmission electron microscopy (cryoHRTEM); see supporting information and Fig. S2.

Nanoparticles characterization.

(Cryo)Transmission Electron Microscopy. CryoTEM images of the nanoparticles were acquired using a Titan Krios 80-300 TEM (Thermo-Fisher Scientific, USA), a microscope optimised for cryoTEM analysis of liquid samples.[1] It is also equipped with an energy filter of model GIF Quantum 968 (Gatan, Inc., USA) located underneath the column to filter out energy-loss electrons and improve contrast in the acquired images. Moreover, behind the GIF column, a highly sensitive direct electron complementary metal oxide semiconductor (CMOS) camera (model K2; Gatan, Inc., USA) was installed to record high-resolution images at extremely low electron-dose conditions (~1 e/Å2). Sample preparation for cryo-TEM analysis was carried out using an automated plunge-freezing tool of the model Vitrobot Mark-IV. Moreover, the specimens were prepared with a special type of copper TEM-grid of model Quatifoil MultiA. These grids have a carbon layer with holes of various sizes and were chosen to vary ice thickness within the holes. In this way, the chance of organic particles being present in the specimen was dramatically higher than with the single hole-size carbon containing grid. Each specimen was prepared by placing 3.5 microliters of solution onto grids, followed by 1 second of blotting time and plunge-freezing into liquid ethane cryogen. The cryo-TEM analysis was performed with the microscope set to an accelerating voltage of 300 kV. Prior to the analysis, the microscope and the GIF were aligned to obtain higher-quality images. Furthermore, the images were recorded under so-called dose-fractionation conditions. In fact, rather than acquiring a single frame with the total electron-beam exposure time, the images were acquired as stacks containing frames with exposure times more than ten times shorter than the total exposure time. The acquired stacks were then aligned and summed along z-direction in order to have final images. This image-recording strategy ensured higher-quality images of organic particles with as little damage as possible. The total electron dose given to images acquired at low magnifications (<50,000X) was kept below 10 e/$Å^2$. Whereas higher-magnification images (>100,000X) received an electron dose of about 20 e/Å2 to maintain good signal-to-noise conditions. The image acquisition and processing were performed using the Gatan Microscopy Suite version 3.2.

Spectroscopic characterisation. The absorption and PL spectra of NP dispersion were recorded in an Agilent Cary 60 UV-Vis and a Cary Eclipse fluorescence spectrometer, respectively.

Microsecond−Second TAS was carried out using a Nd:YAG laser (OPOTEK Opolette 355 II, 6 ns pulse width), which generates UV pulses (355 nm) or visible/NIR pulses (410−2200 nm). A 100 W quartz halogen lamp generates the probe beam, which is sequentially directed through the sample and then through a monochromator before being directed onto a Si photodiode detector (Hamamatsu S1722-01). Pump pulses are directed to the sample through a liquid light guide and are overlapped with the probe beam at the position of the sample. Data acquisition is triggered by scattered laser light using a photodiode. A home-built LabVIEW-based software package was used to acquire the data on two different time scales simultaneously: the microsecond−millisecond signal is sampled using an oscilloscope (Tektronix DPO 3012) after amplification (Costronics 2011 amplifier), whereas the millisecond−second signal is sampled without amplification using a DAQ card (National Instruments USB-6361). Pulse energies were measured using an energy meter (OPHIR Photonics, VEGA P/N 7Z01560). All NPs dispersions were prepared in DI-water, adjusted to equal numbers of absorbed photons, with an absorbance of 0.6 at the excitation wavelength and under anaerobic conditions.

Photoinduced Absorption Spectroscopy (PIAS) was carried out on a modified microsecond-second TAS setup. A high-power LED (Cree XLamp XP-E XPERED-L1-0000-00801, 630 nm) was used as the excitation source and was driven by a high-precision DC power supply (TTi QL564P). The LED is directed to the sample through a liquid light guide. Light pulses are generated via a MOSFET transistor (STMicroelectronics STF8NM50N) and the gate is modulated by the DAQ card (National Instruments, USB-6361). All data were sampled without prior amplification using the DAQ card. Excitation fluences were measured with a digital power meter (Thorlabs PM100), using a silicon photodiode power sensor (Thorlabs S120UV). All NPs dispersions were prepared in DI-water, adjusted to equal numbers of absorbed photons, with an absorbance of 0.6 at the excitation wavelength and under anaerobic conditions.

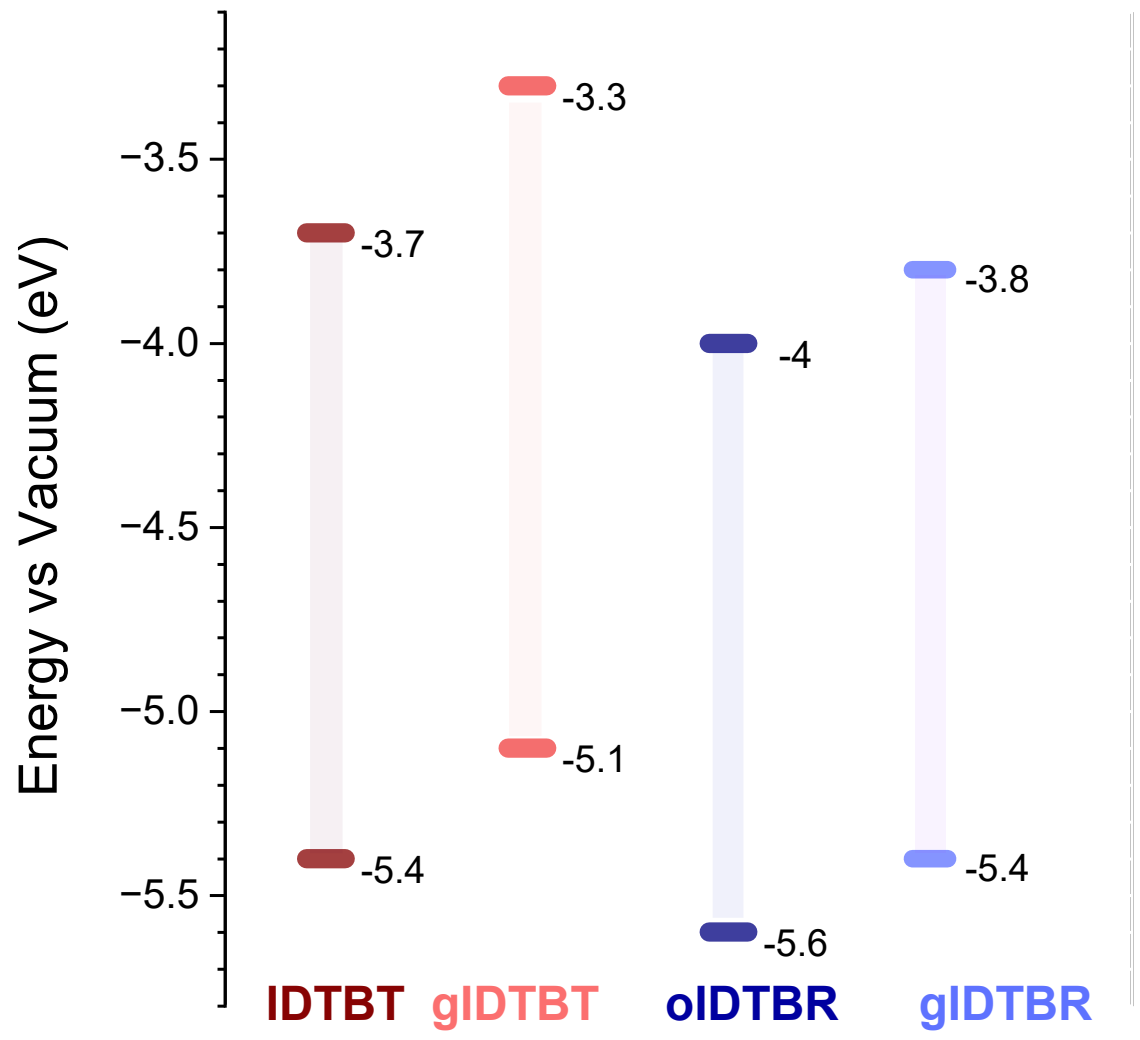

Figure S 1. Energy levels measured using a combination of photoelectron emission spectroscopy in air (PESA) and UV-visible absorption spectroscopy of IDTBT, gIDTBT, oIDTBR, and gIDTBR dry films.

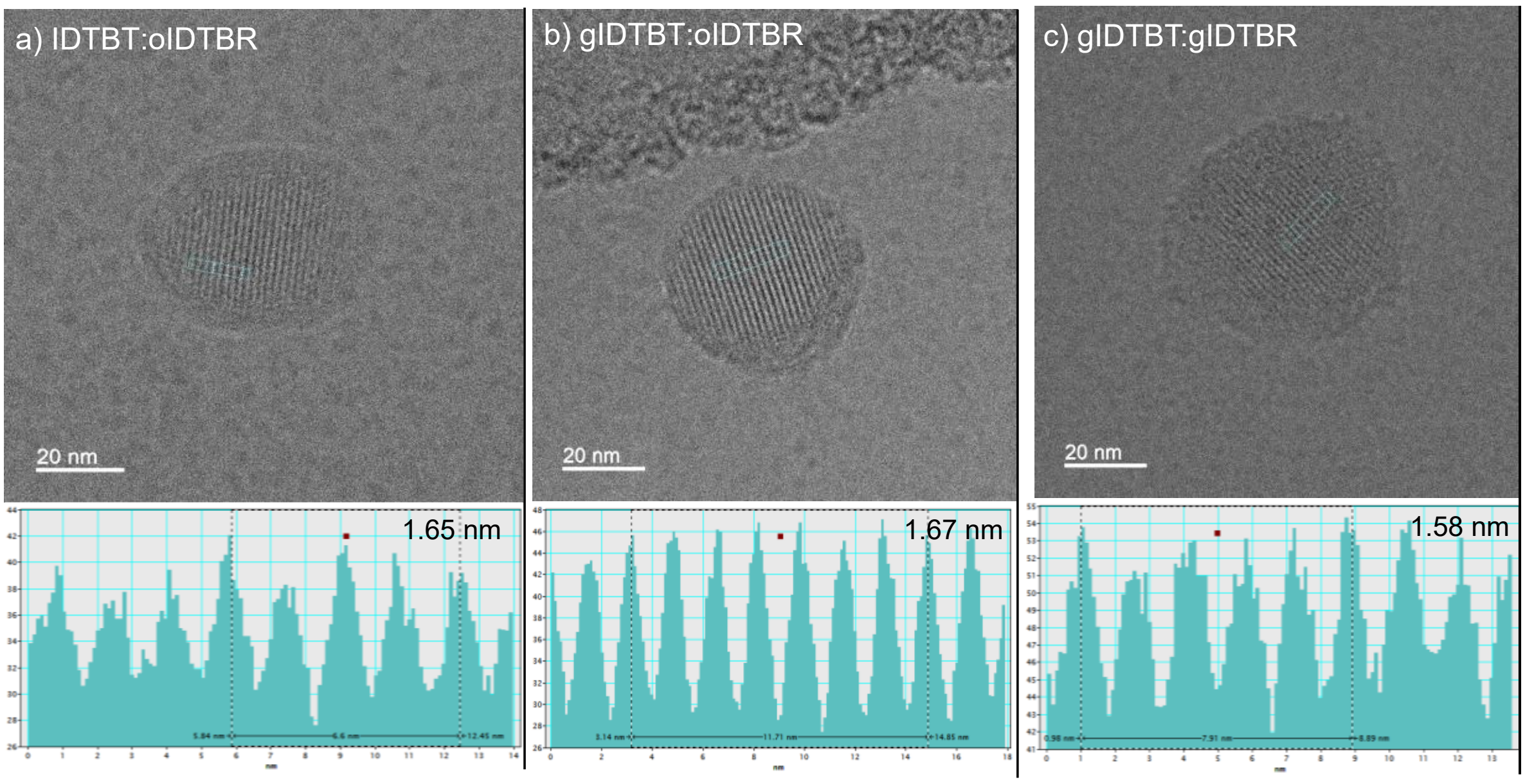

Figure S 2. Comparison of bright-field cryo-HRTEM images (top) and profiles of the periodic spacings highlighted in the rectangles in TEM images (bottom) of a) IDTBT:oIDTBR, b) gIDTBT:oIDTBR and c) gIDTBT:gIDTBR. The periodic lattice spacing (≈1.7 nm) corresponds to the diffraction peak of crystalline oIDTBR. HRTEM images (a,b), previously reported,[1] and are included here as a reference.

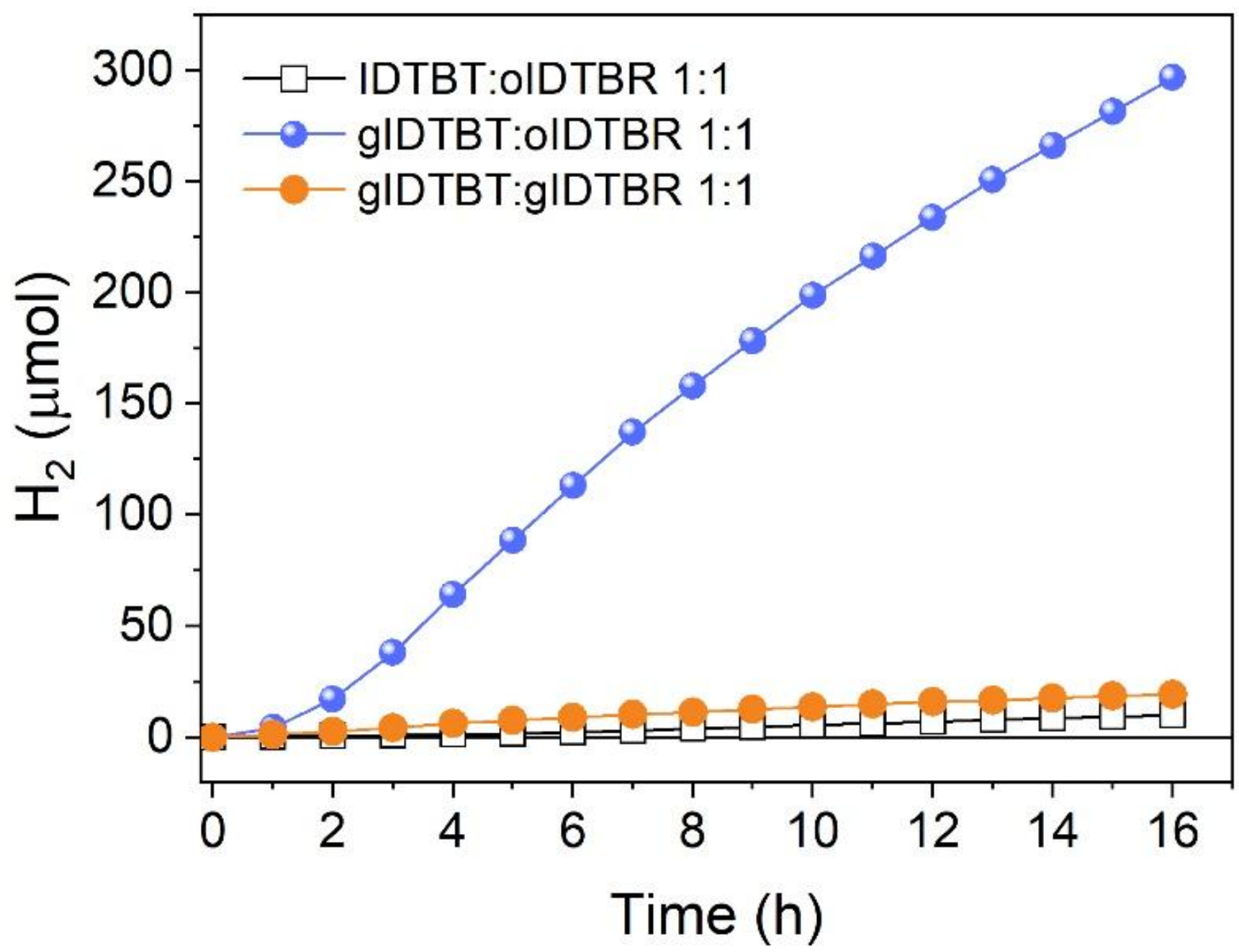

Figure S 3. Comparison of HER of heterojunction NP suspension. Hydrogen evolution conditions: 1 mg NP, 0.2 M AA (12 ml), pH 2, 10 µg (10 wt.%) Pt, AM1.5g solar simulator at 100 $mWcm^{-2}$ (1 sun), 4.41 cm^2 illumination area.

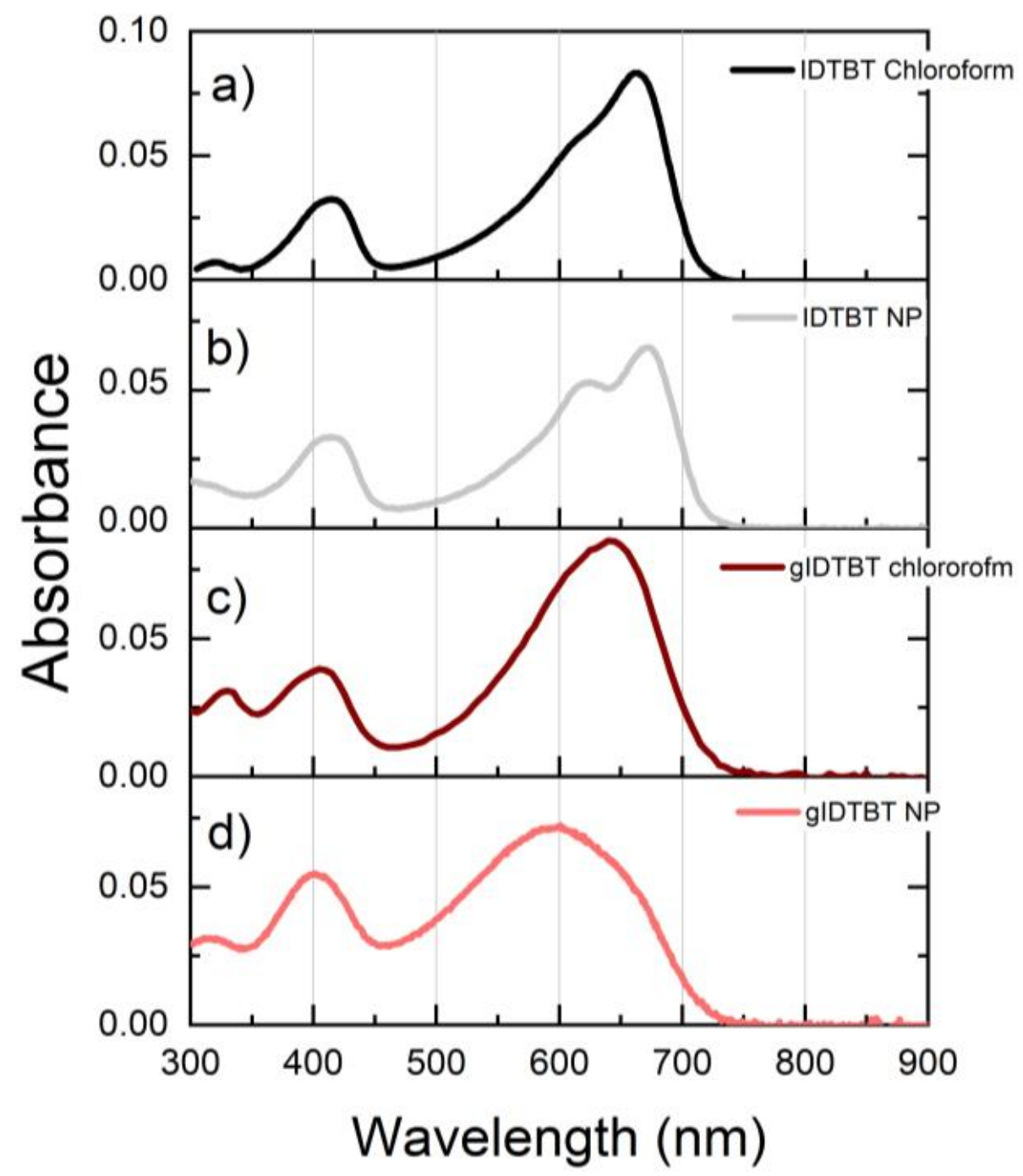

Figure S 4. Comparison of the absorption spectra of neat polymer NP dispersion (water) and the IDTBT and OEG-DTBT in organic solvent (chloroform).

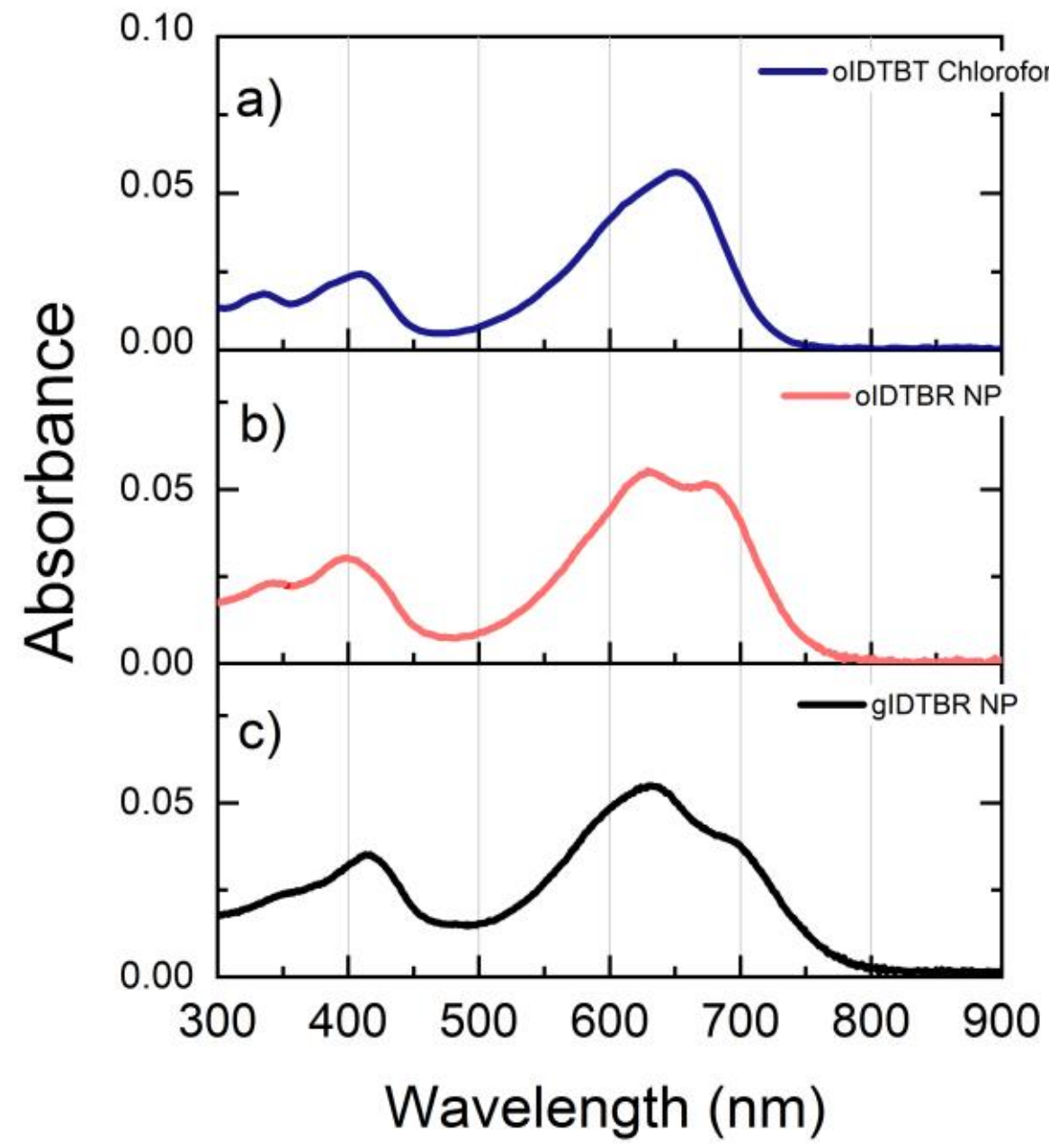

Figure S 5. Comparison of the absorption spectra of the neat NFA NPs dispersion and the g-IDTBR in organic solvent (chloroform).

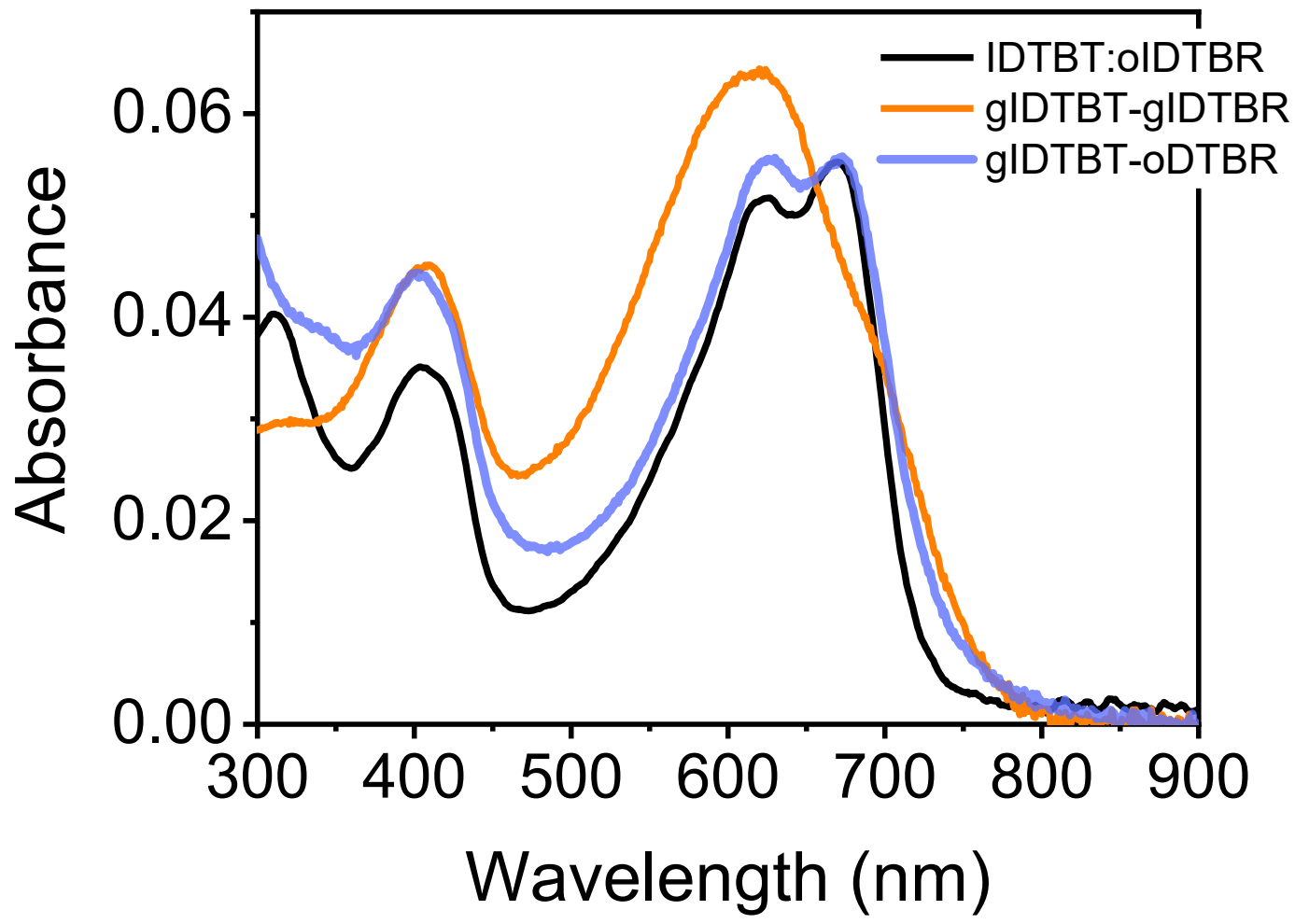

Figure S 6. Comparison of absorption spectra of D:A heterojunction NP dispersion in DI-water. Differences in the ratio between the highest and lowest energy absorption were observed in the heterojunction NPs relative to neat IDTBT, gIDTBT, oIDTBR, and gIDTBR NP dispersions, as a result of D-A domains interactions, which resulted in crystalline differences in the heterojunction NP.

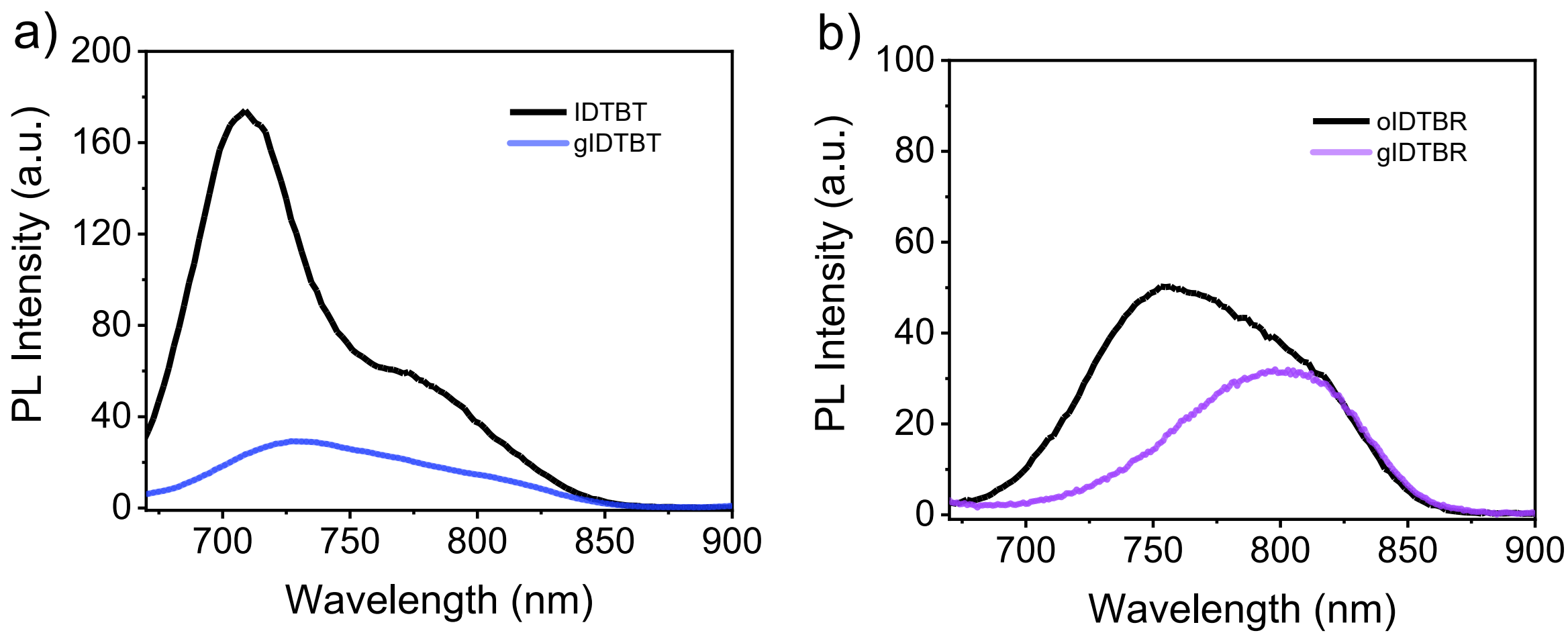

Figure S 7. Comparison of the PL emission spectra in single NP dispersion in water, showing suppressed radiative exciton recombination after OEG-side chain replacement in a) IDTBT polymer and b) oIDTBR NP acceptor dispersion. Excitation wavelength 630 nm. The NP dispersion was adjusted to the same absorbed photons.

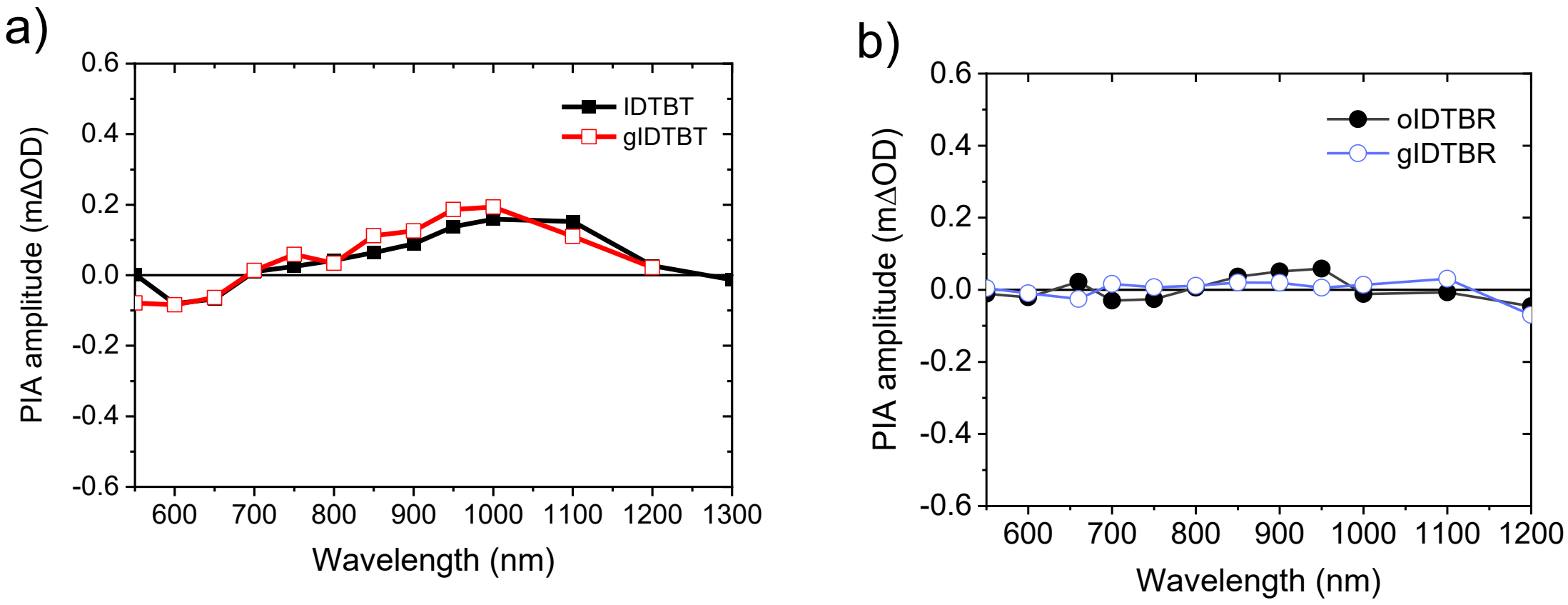

Figure S 8. a) Comparison of PIAS spectra of neat a) polymers NP and b) NFA NPs dispersion in DI-water. Recorded at 4 seconds of continuous LED illumination (630 nm, 20 mW cm^{-2})

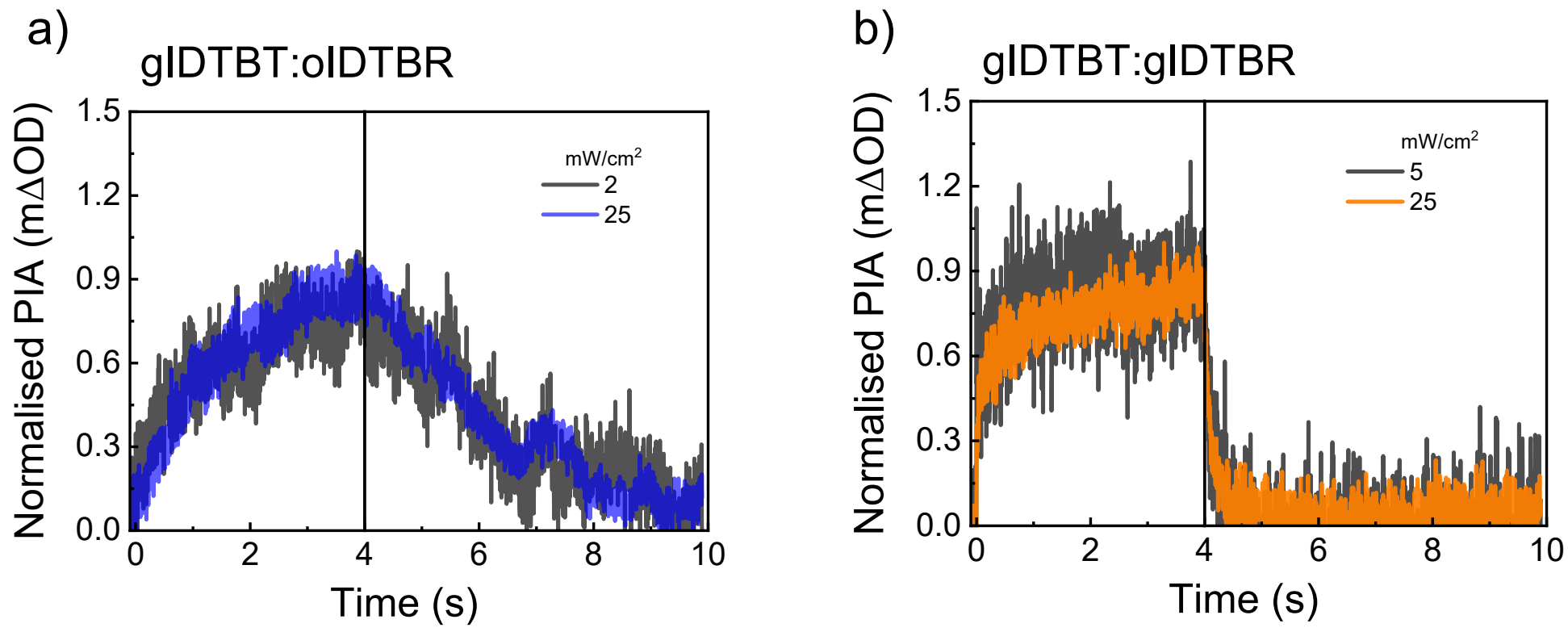

Figure S 9. Comparison of normalised PIAS kinetics of the heterojunction NP dispersion at the lowest and highest LED fluence, showing fluence-independent kinetic decay.

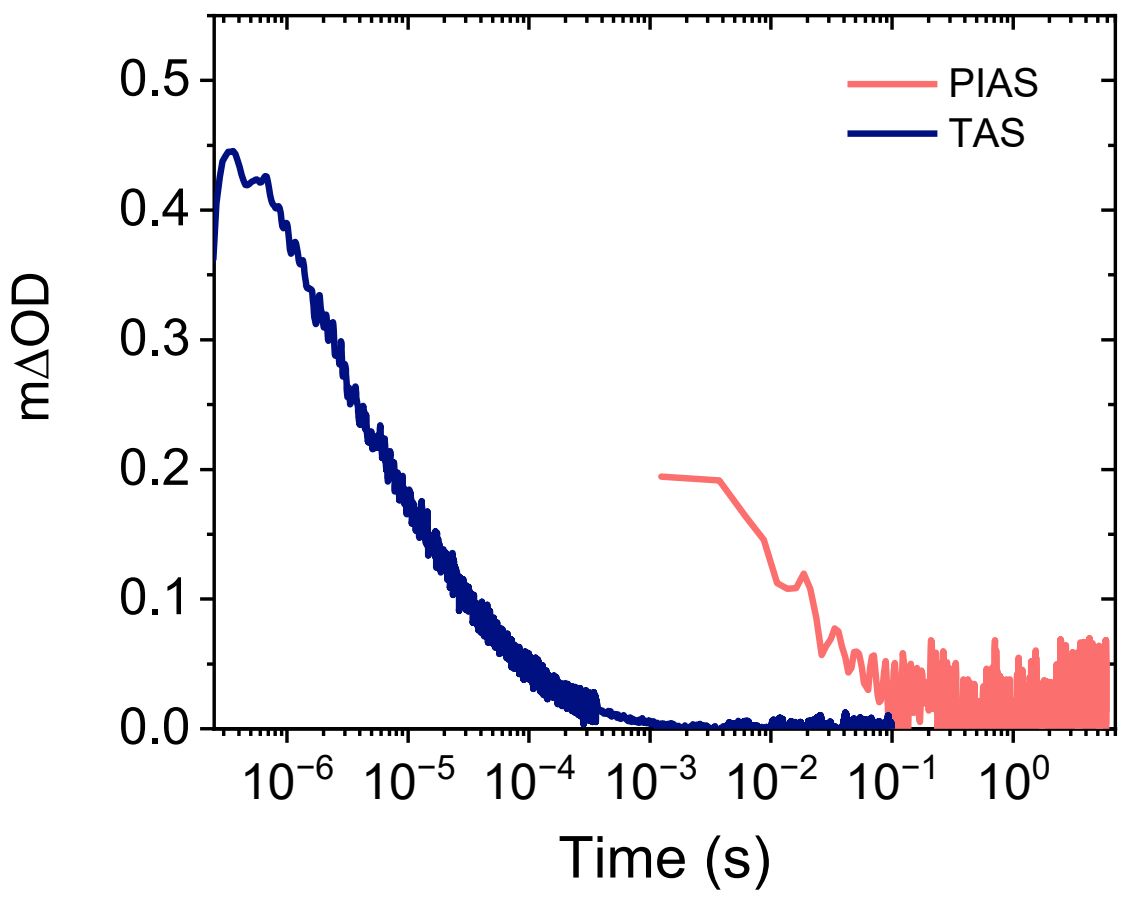

Figure S 10. Comparison of TAS and PIAS traces (probed at 1000 nm) of gIDTBT:gIDTBR NP dispersion in water under anaerobic conditions. TAS: pulsed excitation at 650 nm (290 μJ cm⁻²). PIAS: LED illumination at 630 nm (20 mW cm⁻²).

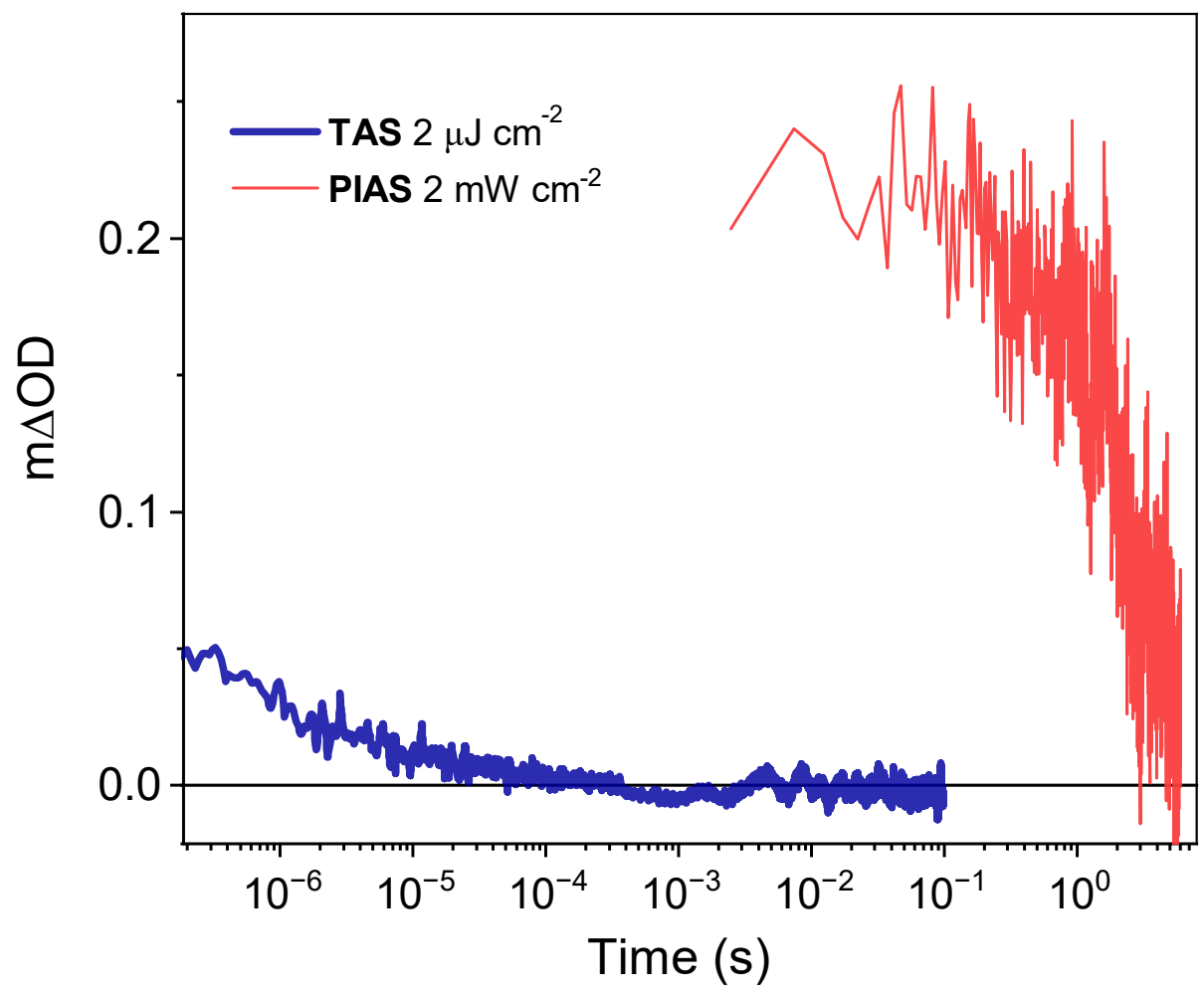

Figure S 11. Comparison of TAS and PIAS traces (probed at 1000 nm) of gIDTBT:oIDTBR NP dispersion in water under anaerobic conditions at the lowest excitation density explored.

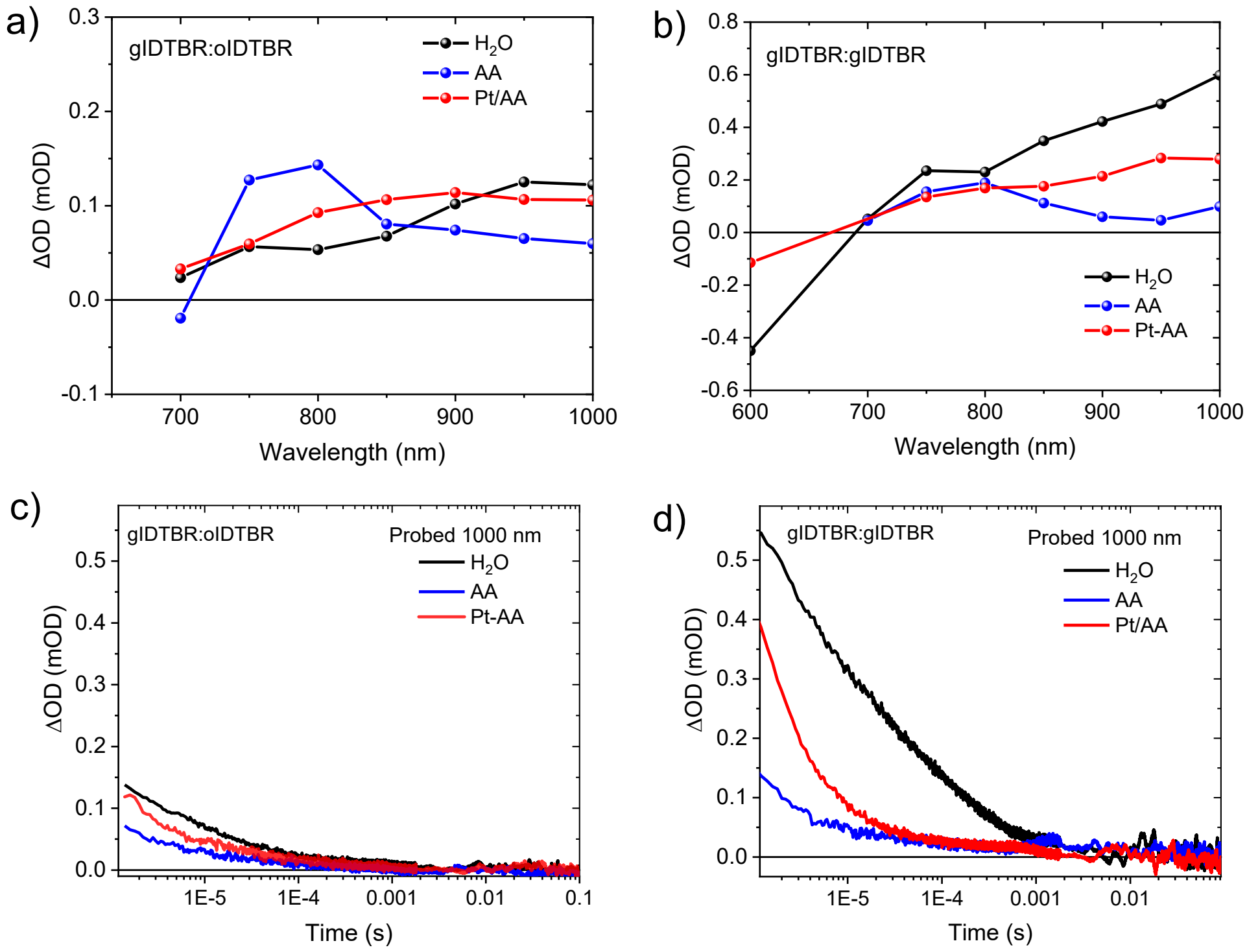

Figure S 12. Comparison of a-b) the TA spectra of OEG-heterojunction NPs recorded at a 2 µs decay time and different probing wavelengths in water and with AA and Pt-AA. c-d) Comparison of the TA kinetics (probed at 1000 nm) of heterojunction NP dispersion in water and with AA and Pt-AA. Laser 650 nm, 200 µJ cm^{-2}). The TAS measurements show that the addition of a hole scavenger (0.2 M AA) reduces signals associated with photogenerated holes while enhancing those of photogenerated electrons, confirming that the observed spectral evolution is primarily attributed to the recombination of $IDTBT^{+}$ charges.

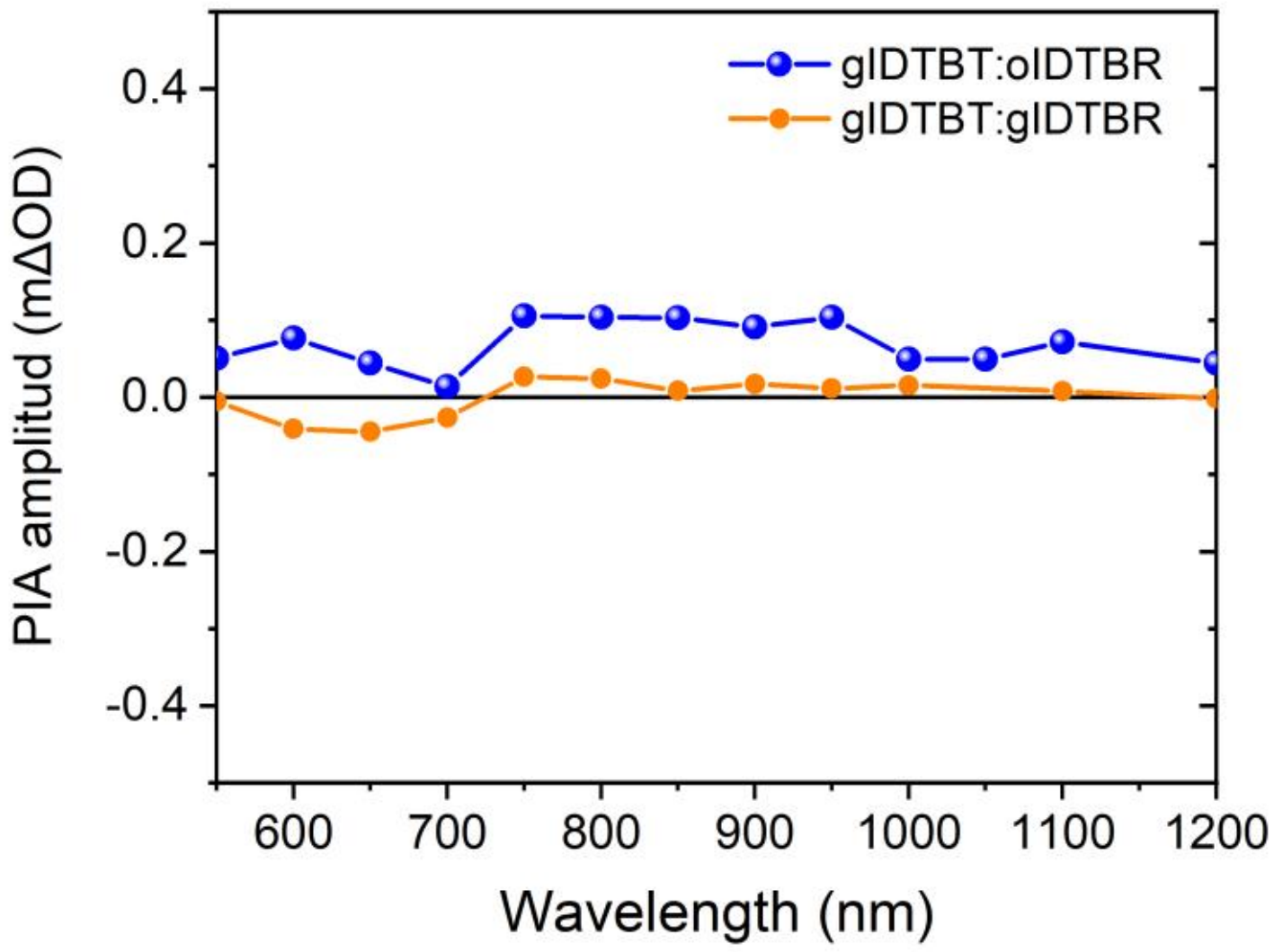

Figure S 13. Comparison of PIAS spectra of gIDTBT:oIDTBR and gIDTBT:gIDTBR heterojunction NP dispersions in the presence of Pt and ascorbic acid (AA, 0.2 M), showing substantially reduced charge accumulation compared with the corresponding measurements in the absence of Pt and AA (Figure 3). The spectral differences may reflect changes in the relative contributions of photoinduced charge species under these reaction conditions. Spectra were recorded after 4 s of continuous LED illumination (630 nm, 20 mW cm^{-2}).

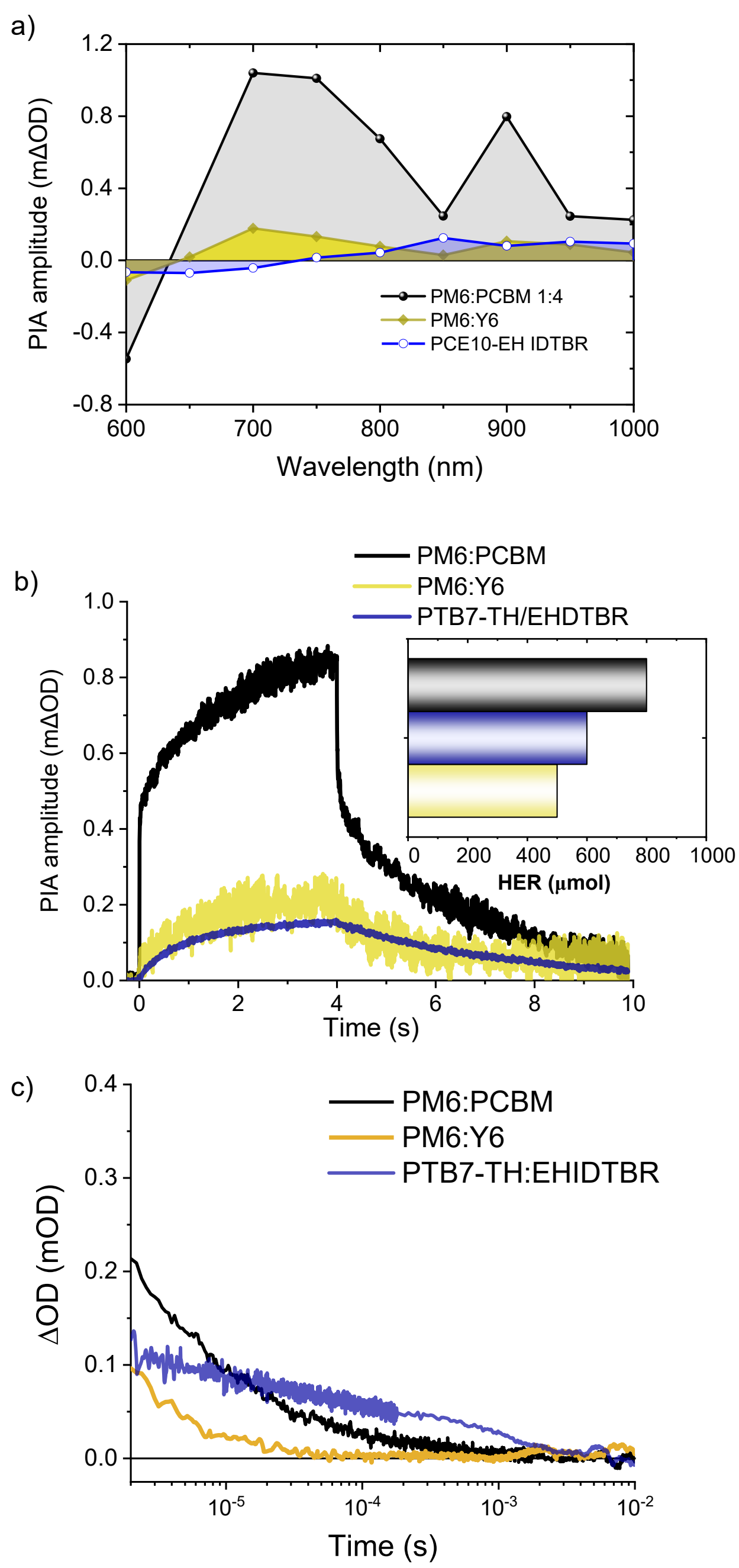

Figure S 14. a) Comparison of PIAS spectra of D/A heterojunction NP dispersion. b) Comparison of PIAS kinetics of heterojunction dispersion, probed at 700 nm for PM6:PCBM (1:4) and PM6:Y6 (7:3) and at 850 nm for PTB7-TH:EHIDTBR (3:7). Inset: Average hydrogen evolution rates of the D/A heterojunction at 16 h, using 1 mg photocatalyst, 12 ml of 0.2 mol l-1 AA, and Pt loadings of 10%. NPs previously reported in the literature. [2,3] PIAS measurements were recorded after 4 seconds of continuous LED illumination (630 nm, 20 mW cm−2). The PIAS spectra primarily showed the absorption signal of the $PM6^{+}$ polaron. **c)** Comparison of the TA kinetics of heterojunction NP dispersion, probed at 700 nm for PM6:PCBM (1:4) and PM6:Y6 (7:3) and at 850 nm for PTB7-TH:EHIDTBR (3:7). TAS Excitation wavelength 650 nm (200 μJ cm^{-2}). These TAS traces are provided for comparison; detailed investigations of the charge-carrier dynamics and their excitation-intensity dependence in these systems have been reported previously.[3,4]

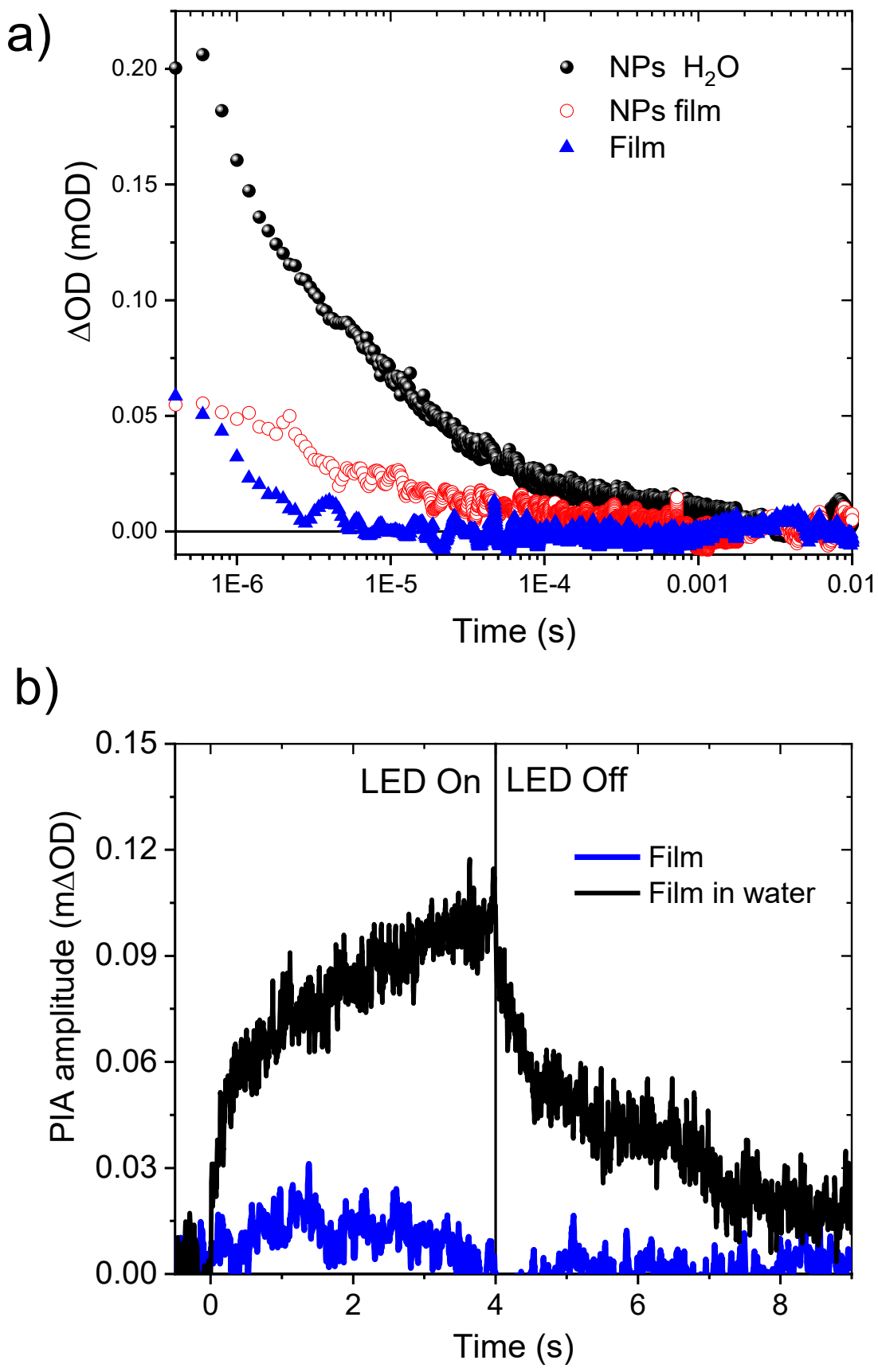

Figure S15. a) Comparison of transient absorption decay dynamics in gIDTBT:oIDTBR heterojunction NPs dispersion and films probed at 1000 nm, assigned to IDTBT polaron decay. Excited at 650 nm (fluence: 225 µJ cm^{-2}). b) PIAS kinetic of dry heterojunction film gIDTBT:oIDTBR and soaked in water probed at 1000 nm. LED illumination (630 nm, 20 mW cm^{-2}) during 4 s, in N_2 atmosphere.

References.

1 J. Kosco, S. Gonzalez-Carrero, C. T. Howells, W. Zhang, M. Moser, R. Sheelamanthula, L. Zhao, B. Willner, T. C. Hidalgo, H. Faber, B. Purushothaman, M. Sachs, H. Cha, R. Sougrat, T. D. Anthopoulos, S. Inal, J. R. Durrant and I. McCulloch, *Advanced Materials*, 2022, **34**, 2105007.

2 J. Kosco, S. Gonzalez-Carrero, C. T. Howells, T. Fei, Y. Dong, R. Sougrat, G. T. Harrison, Y. Firdaus, R. Sheelamanthula, B. Purushothaman, F. Moruzzi, W. Xu, L. Zhao, A. Basu, S. De Wolf, T. D. Anthopoulos, J. R. Durrant and I. McCulloch, *Nature Energy*, 2022, **7**, 340–351.

3 J. Kosco, M. Bidwell, H. Cha, T. Martin, C. T. Howells, M. Sachs, D. H. Anjum, S. Gonzalez Lopez, L. Zou, A. Wadsworth, W. Zhang, L. Zhang, J. Tellam, R. Sougrat, F. Laquai, D. M. DeLongchamp, J. R. Durrant and I. McCulloch, *Nat. Mater.*, 2020, **19**, 559–565.

4 S. Gonzalez-Carrero, J. Kosco, T. Fei, I. McCulloch and J. R. Durrant, *Chem. Sci.*, 2024, **15**, 19044–19056.
